# Crosstalk between KDEL receptor and EGF receptor mediates cell proliferation and migration via STAT3 signaling

**DOI:** 10.1186/s12964-024-01517-w

**Published:** 2024-02-20

**Authors:** Jie Jia, Lianhui Zhu, Xihua Yue, Shuocheng Tang, Shuaiyang Jing, Chuanting Tan, Yulei Du, Jingkai Gao, Intaek Lee, Yi Qian

**Affiliations:** 1 School of Life Science and Technology, ShanghaiTech University, Pudong, Shanghai, China; 2 Present address: Department of Pharmacology, School of Medicine, Southern University of Science and Technology, Shenzhen, China

**Keywords:** KDEL receptor, EGF receptor, STAT3, GRP78, ERp57, Golgi, Endosomes, Clathrin

## Abstract

Hostile microenvironment of cancer cells provoke a stressful condition for endoplasmic reticulum (ER) and stimulate the expression and secretion of ER chaperones, leading to tumorigenic effects. However, the molecular mechanism underlying these effects is largely unknown. In this study, we reveal that the last four residues of ER chaperones, which are recognized by KDEL receptor (KDELR), is required for cell proliferation and migration induced by secreted chaperones. By combining proximity-based mass spectrometry analysis, split venus imaging and membrane yeast two hybrid assay, we present that EGF receptor (EGFR) may be a co-receptor for KDELR on the surface. Prior to ligand addition, KDELR spontaneously oligomerizes and constantly undergoes recycling near the plasma membrane. Upon KDEL ligand binding, the interactions of KDELR with itself and with EGFR increase rapidly, leading to augmented internalization of KDELR and tyrosine phosphorylation in the C-terminus of EGFR. STAT3, which binds the phosphorylated tyrosine motif on EGFR, is subsequently activated by EGFR and mediates cell growth and migration. Taken together, our results suggest that KDELR serves as a *bona fide* cell surface receptor for secreted ER chaperones and transactivates EGFR-STAT3 signaling pathway.

**Supplementary Information:**

The online version contains supplementary material available at 10.1186/s12964-024-01517-w.

## Introduction

Endoplasmic reticulum (ER) chaperones have been well-studied for their roles in assisting folding of nascent proteins. In cancer cells, due to high growth rate, hypoxia, nutrient deficiency, and acidic microenvironment, capacity of ER protein folding machinery is overwhelmed by the large amount of unfolded and misfolded proteins, which triggers an unfolded protein response (UPR) and induces the expression of ER chaperones to improve ER protein folding capacity [1, 2]. Unexpectedly, recent studies have demonstrated that several ER chaperones, including the 78-kD glucose-regulated protein (GRP78) and the endoplasmic reticulum resident protein 57 (ERp57), are not only transcriptionally up-regulated, but also secreted and bound to the cell surface in an unknown manner to facilitate cell proliferation and migration [3–7]. However, it remains elusive which plasma member protein(s) serves as their receptor(s).

As with most soluble ER chaperone proteins, both GRP78 and ERp57 contain a KDEL-like motif at their extreme carboxyl terminus, which is recognized by KDEL receptor (KDELR) for their retrieval back to the ER from the Golgi apparatus by a coat protein I (COPI)-dependent pathway to maintain the dynamic homeostasis between ER and the Golgi [8–11]. KDELR is a seven transmembrane protein and mostly localized to the Golgi at steady state. Interestingly, recent findings have indicated that a subgroup of KDELR resides on the plasma membrane, circulates between the cytomembrane and the Golgi, and contributes to the cell surface binding of ER chaperones, suggesting that KDELR may serve as a receptor for secreted ER chaperones on the cell membrane with an unknown downstream signaling network [12–17].

Although surface-expressed KDELR undergoes endocytosis via clathrin-coated vesicles, it does not contain a recognition motif for cargo-selective clathrin adaptor protein complex AP-2 [18, 19]. Therefore, it is likely that KDELR utilizes a cell surface co-receptor for its endocytic pathways via clathrin. In our previous study, we used an in vivo tagging strategy to label proteins in proximity to KDELR and found EGF receptor (EGFR) as a previously unreported potential KDELR-interacting protein [20]. The epidermal growth factor receptor (EGFR) is one of the best known plasma membrane receptors and has been extensively investigated for decades as a cancer therapeutic target [21–23]. It is composed of a ligand-binding extracellular domain, a transmembrane span, a cytoplasmic tyrosine kinase domain, and a carboxyl tail. Upon a ligand binding to its extracellular domain, EGFR forms homo- or hetro-dimers, leading to its activation by auto- or trans-phosphorylation of certain tyrosine residues in the cytoplasmic tail and subsequent endocytosis [24–26]. Internalized receptors provide cytoplasmic phosphotyrosine residues as docking sites for interacting proteins and recruit downstream partners, including adaptors, kinases, and phosphatases to modulate a variety of signaling pathways for cell proliferation, differentiation, and migration [27, 28]. Around twenty tyrosine residues in the cytoplasmic region of EGFR have been reported to each bind one or more interacting partners and accumulate over one hundred proteins as EGFR interacting proteins, implying the diversity and complexity of the EGFR interactome [29–31].

In the present work, we sought to dissect the role of KDELR-EGFR interaction at the PM and the detailed mechanisms of secreted ER chaperones transactivating EGFR via KDELR. Our results indicate that the last four amino acids of ER chaperones, including GRP78, MANF, and ERp57, are required for chaperone-induced cell growth and migration. Further, we provide evidences that KDEL-KDELR binding on cell surface transactivates EGFR, resulting in the co-internalization of KDELR and EGFR in clathrin-coated vesicles (CCVs), trafficking to the Golgi and recycling back to the plasma membrane. Finally, phosphorylation of EGFR induced by KDEL peptide activates a transcriptional factor, STAT3, leading to cell proliferation and migration.

## Results

### KDEL-like motif is essential for ER protein-induced cell proliferation and migration

Increasing evidence has shown that secreted ER proteins are important for cell survival, growth, and progression in various cancer cell lines [3, 4, 6, 32, 33]. However, it is currently unknown that whether the common receptor for ER chaperones, KDELR, is involved in mediating these functions. There are three isoforms of KDELR (KDELR1, 2, and 3), all of which appear to expressed on the cell surface in mammalian cells [15]. Since KDELR1 is the most studied and best representative of the family, we focus on KDELR1 and refer to it as KDELR in the rest of this study.

As the last four amino acids of ER proteins are essential for KDELR binding, we purified recombinant GRP78, both wildtype and KDEL-deleted mutant (GRP78ΔKDEL), from E. coli and used them in cell counting (CCK-8) and wound healing experiments to investigate whether KDEL motif influences cell proliferation and migration (Sup. Figure 1A).

**Fig. 1:**
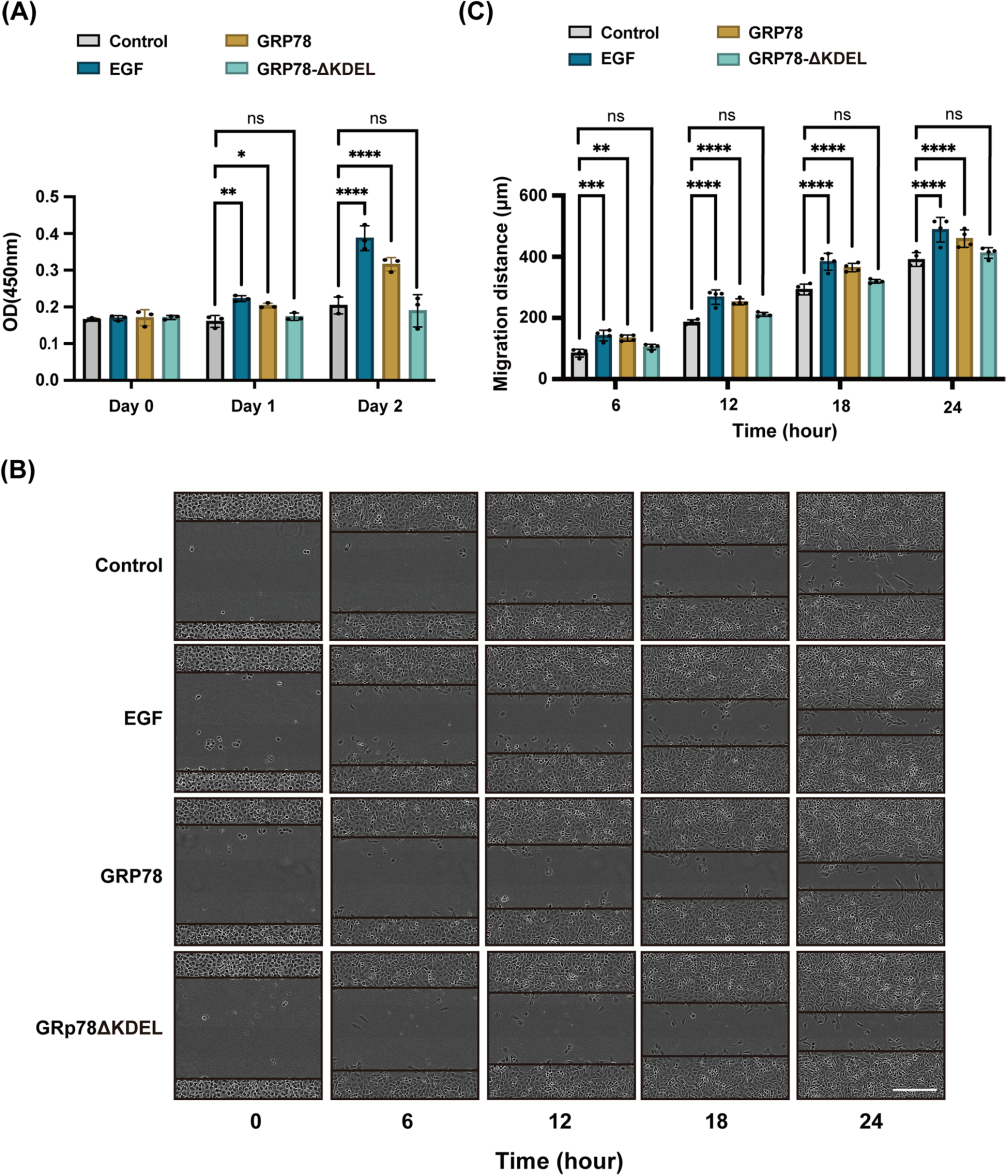
GRP78 stimulates cell proliferation and migration via its KDEL motif. HT1080 cells were incubated with DMSO, 30 nM EGF, 50 nM GRP78 or GRP78ΔKDEL, for evaluating the effects of GRP78 on cell growth and migration. **A** EGF and GRP78 induced cell proliferation. Cell viability was measured by CCK-8 assay on day 0, 1, and 2 after incubation with DMSO or indicated proteins in MEM supplemented with 10% FBS. Histogram summarizes the OD450 measurements of cells at indicated time points. Statistical analysis was performed using two-way ANOVA with a Dunnett’s post-hoc test for multiple comparisons. *n* = 3 independent experiments. **B**,** C** EGF and GRP78 enhanced cell migration during wound healing. Phase contrast images of cells incubated with DMSO, or indicated protein supplements at 6, 12, 18, and 24 h after wounding (**B**). The migration distance in wound healing was statistically analyzed using two-way ANOVA with a Dunnett’s post-hoc test for multiple comparisons (**C**). *n* = 4 independent experiments. For all graphs, data are presented as mean ± SD. *: *P* < 0.05. ***: *P* < 0.001. ****: *P* < 0.0001. ns: not significant. Scale bar = 300 μm

For the cell proliferation (CCK-8) assay, HT1080 cells were seeded on a 96-well plate and incubated with EGF, GRP78, or GRP78ΔKDEL. The proliferation rate was evaluated by the optical density (OD 450 nm) measurements on 0, 1, and 2 days after seeding. As shown in Fig. 1A, EGF and GRP78-incubated cells showed a significantly higher growth rate than control cells, whereas GRP78ΔKDEL-treated cells revealed similar proliferation rate as control cells. A peptide that contains the last seven amino acids (TAEKDEL) of GRP78 promoted HeLa cell growth in a similar manner, but to a less extent (Sup. Figure 1B). These results indicated that the recombinant ER protein, GRP78, induce cell proliferation, which is probably dependent on their binding to surface expressed KDELR.

Next, we tested cell migration rate during the wound-healing process. HT1080 cells were seeded onto a 96-well plate and grown until full confluency before cells were scratched and incubated with EGF, GRP78, and GRP78ΔKDEL. Images of wound closure were captured at 6, 12, 18, and 24 h by phase contrast microscopy and the migration distance was measured using ImageJ software. Strikingly, GRP78-treated cells were observed to migrate at a higher rate than control cells on both day 1 and day2, but GRP78ΔKDEL had limited effect (Fig. 1B and C), suggesting that the last four amino acids are essential for GRP78-stimulated cell migration.

Another KDELR ligand protein, mesencephalic astrocyte-derived neurotrophic factor (MANF), has been described to promote cell differentiation, migration, and regeneration as well [17, 34, 35]. We investigated whether the KDEL-like motif on MANF was required for its function using HeLa cells. The results of CCK-8 assay showed that recombinant MANF, but not MANFΔRTDL, induced cell proliferation (Sup. Figure 1C and D). Cell migration rate was evaluated by the transwell experiments. HeLa cells growing on the upper layer were induced with EGF, MANF or MANFΔRTDL for 18 h. Then the cells that migrated through permeable membrane were stained with crystal violet. The results showed that purified His-tagged MANF induced significant cell migration while MANFΔRTDL did not (Sup. Figure 1E and F). Taken together, our results suggested that cell surface KDELR is the receptor for secreted ER proteins-induced cell proliferation and migration.

### KDELR on the plasma membrane does not interact with G_α_ proteins

KDELR appears to serve as a G protein-coupled receptor (GPCR) at the Golgi by binding α subunits of heterotrimeric G proteins and activating their downstream signaling pathways [36–39]. Therefore, we first examined whether cell surface localized KDELR behaves like a GPCR and binds to G_α_ proteins using bimolecular fluoresecence complementation (BiFC) assay [40, 41]. As illustrated in Sup. Figure 2A, the N-terminal half of a fluorescent protein, Venus, and a flag tag were fused to the C-terminus of Halo-KDELR that contains a signal peptide as a membrane insertion signal (Halo-KDELR-flag-VN). All G_α_ proteins were individually subcloned to upstream of a myc linker and the C-terminal half of Venus (G_α_-myc-VC). It is well known that heterotrimeric G protein interacts with GPCR in the absence of a signal, when G_α_ is bound to guanosine diphosphate (GDP). Upon agonist activation, the receptor serves as a guanine nucleotide exchange factor (GEF) for the G_α_ subunit, leading to dissociation of active G_α_-GTP and GPCR. Thus, we also introduced a single amino acid mutation in G_α_ proteins (G_αs_ S54C, G_αq_ S53C, G_αo_ S47C) to mimic their GDP-bound state (G_α_-GDP-myc-VC) for improved binding with KDELR. HeLa cells were co-transfected with Halo-KDELR-flag-VN and G_α_-myc-VC, or G_α_-GDP-myc-VC for 18 h, followed by confocal microscope analysis. Venus signal was not detected on cell membrane in our experiments, suggesting that KDELR may not function as a GPCR on plasma membrane (Sup. Figure 2B and C).

### EGFR and TfR are identified as potential interacting proteins of surface KDELR

Our previous study showed that cell surface KDELR is able to be internalized through clathrin-mediated endocytic pathway [15]. However, it lacks the canonical dileucine-based sorting signal for clathrin adaptor protein 2 (AP-2), which is responsible for cargo recognition [19, 42]. Therefore, it is likely that KDELR has a co-receptor on the plasma membrane for its endocytosis and signaling. Although EGFR-STAT3 signaling is the most frequently reported downstream pathway of surface-bound ER proteins, several other pathways may be involved as well. For example, MANF has been described to activate platelet-derived growth factor (PDGF)-like signaling in the retina of flies and mice [35].

**Fig. 2:**
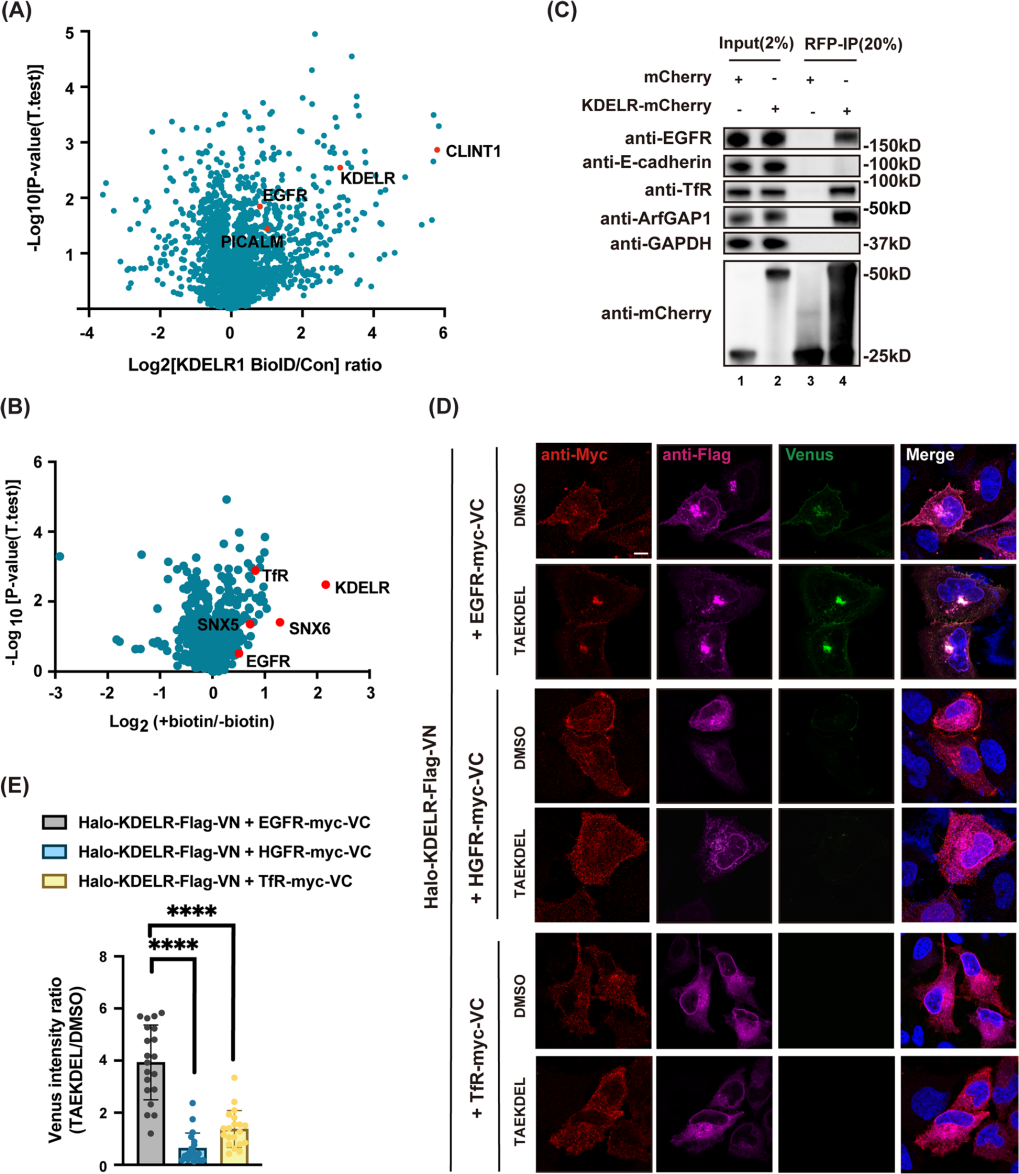
EGFR was identified as a novel KDELR-interacting protein. **A** Volcano plot representing results of KDELR BioID. HeLa cells were transfected with KDELR-BirA^*^ or KDELR-myc for 24 h and incubated with 50 μM biotin for 6 h, prior to cell disruption. Proteins in close proximity to KDELR- BirA^*^ were enriched by streptavidin beads and subjected to mass spec analysis. The logarithmic ratios of protein intensities identified in KDELR-BirA^*^ versus control (KDELR-myc) were plotted against negative logarithmic *p*-value of the t test performed from triplicate experiments. KDELR, surface receptor and clathrin-related proteins are marked in red. **B** Volcano plot showing results of surface-KDELR pulldown-mass spec experiment. Halo-KDELR expressing on the cell surface of HeLa cells were labeled with non-membrane permeable biotin-conjugated Halo ligand. Proteins in cell lysates were precipitated by streptavidin beads and analyzed by mass spec. The logarithmic ratios of protein intensities in HeLa cells with biotin-conjugated Halo ligand incubation versus DMSO were plotted against negative logarithmic *p*-value of the t test performed from triplicate experiments. **C** Co-IP experiments in HeLa cells overexpressing mCherry or KDELR-mCherry using anti-RFP beads confirmed that EGFR and TfR as KDELR-interacting proteins. **D**,** E** The interaction between EGFR and KDELR brought two halves of Venus protein together to generate Venus signals. HeLa cells transfected with Halo-KDELR-Flag-VN and EGFR-myc-VC, HGFR-myc-VC, or TfR-myc-VC were treated with 50 μM TAEKDEL peptide or DMSO at 4 °C for 30 min, followed by incubation at 37 °C for 30 min. Cells were then fixed, stained with indicated antibodies, and observed under confocal microscope (D). Venus signal ratio of TAEKDEL- versus DMSO-treated was statistically analyzed by one-way ANOVA with Tukey’s multiple comparisons (E). *n* = 20 cells pooled from 3 independent experiments. ****: *P* < 0.0001. Scale bar = 10 μm

To identify the potential co-receptor of surface KDELR, we revisited our previous data of KDELR interactome identified in the mass spectrometry of BioID experiment, based on protein proximity to C-terminally biotin ligase-fused KDELR (KDELR-BirA^*^) [20]. In this database, we found a plasma membrane receptor, EGFR, and two clathrin-related proteins, clathrin interactor 1 (CLINT1) and phosphatidylinositol binding clathrin assembly protein (PICALM), suggesting that EGFR may bind intracellular KDELR (Fig. 2A).

Since most of KDELR-BirA^*^ resides at the Golgi apparatus, our BioID assay is expected to identify KDELR-interacting proteins close to the Golgi. To selectively study the interactome of the surface-expressed KDELR, we transfected HeLa cells with previously used N-terminally Halo-tagged KDELR (Halo-KDELR, [15]), selectively labeled cell surface KDELR with non-membrane permeable biotin-conjugated Halo ligands in living cells, and purified KDELR-associated proteins with streptavidin agarose, prior to mass spectrometry analysis. This experiment revealed proteins involved in membrane trafficking, such as sorting nextin 5 and 6 (SNX5 and SNX6), as well as two surface receptors, EGFR and transferrin receptor (TfR) (Fig. 2B).

Next, we tested whether the receptors identified in mass spectrometry experiments appeared in CCVs as the recycling of KDELR between cell surface and the Golgi was shown to be mediated by clathrin [15]. To include a negative control in our experiments, we took advantage of a previous study showing that a KDELR mutant, D91A/T92A, causes complete ER retention [43]. Since this mutant is not able to reach the Golgi, it will not be found in purified CCVs. We transfected HeLa cells with Halo-KDELR or Halo-KDELR D91A/T92A mutant and purified CCVs from HeLa cells according to a published protocol [44]. As expected, western blots analysis using specific antibodies indicated that clathrin and the μ1 subunit of clathrin adaptor protein 1 (AP1M1), but not the β subunit of COPI (β-COP), were enriched in CCVs obtained from both wildtype and mutant KDELR transfected cells. EGFR and TfR were also accumulated in CCVs from both kinds of cells, consistent with the fact that they are constantly internalized and recycled even in the absence of their ligands [45, 46]. On the other hand, only wildtype KDELR was found in CCV fractions, suggesting that internalized KDELR may be transported in CCVs (Sup. Figure 3A).

To further confirm that EGFR and TfR are interacting receptors for KDELR, we performed the immunoprecipitation (IP) experiment using anti-red fluorescent protein (anti-RFP) beads in HeLa cells transfected with mCherry or KDELR-mCherry, and in endogenously Flag-mCherry tagged KDELR knockin cells [20]. KDELR-bound proteins were analyzed by western blots. EGFR, TfR, and ArfGAP1, which is a known KDELR binding protein, were pulled down by KDELR, while cell surface proteins, E-cadherin and integrin α2, were not found in the co-immunoprecipitated fraction (Fig. 2C, Sup. Figure 3B).

### EGFR is a novel KDELR’s interacting protein

Since EGFR has been reported to bind TfR, it is difficult to determine which receptor, EGFR or TfR, is the co-receptor on cell surface for KDELR using conventional methods [47]. Therefore, we performed proximity-based BiFC assays of KDELR with EGFR, TfR and the hepatocyte growth factor receptor (HGFR, a negative control). HeLa cells were co-transfected with Halo-KDELR-Flag-VN and EGFR-, HGFR- or TfR-myc-VC overnight, prior to incubation with DMSO or TAEKDEL peptide for 30 min. Confocal images showed that Venus signal was detected on cell surface and at the Golgi only in cells co-expressing Halo-KDELR-Flag-VN with EGFR-myc-VC, but not TfR-myc-VC, or HGFR-myc-VC. No background signals were observed when individual construct was transfected in cells (Sup. Figure 3C and D). Moreover, the Venus signal generated by EGFR-KDELR association was greatly enhanced after TAEKDEL peptide addition, indicating the interaction between EGFR and KDELR was improved by KDEL-KDELR binding (Fig. 2D and E).

**Fig. 3:**
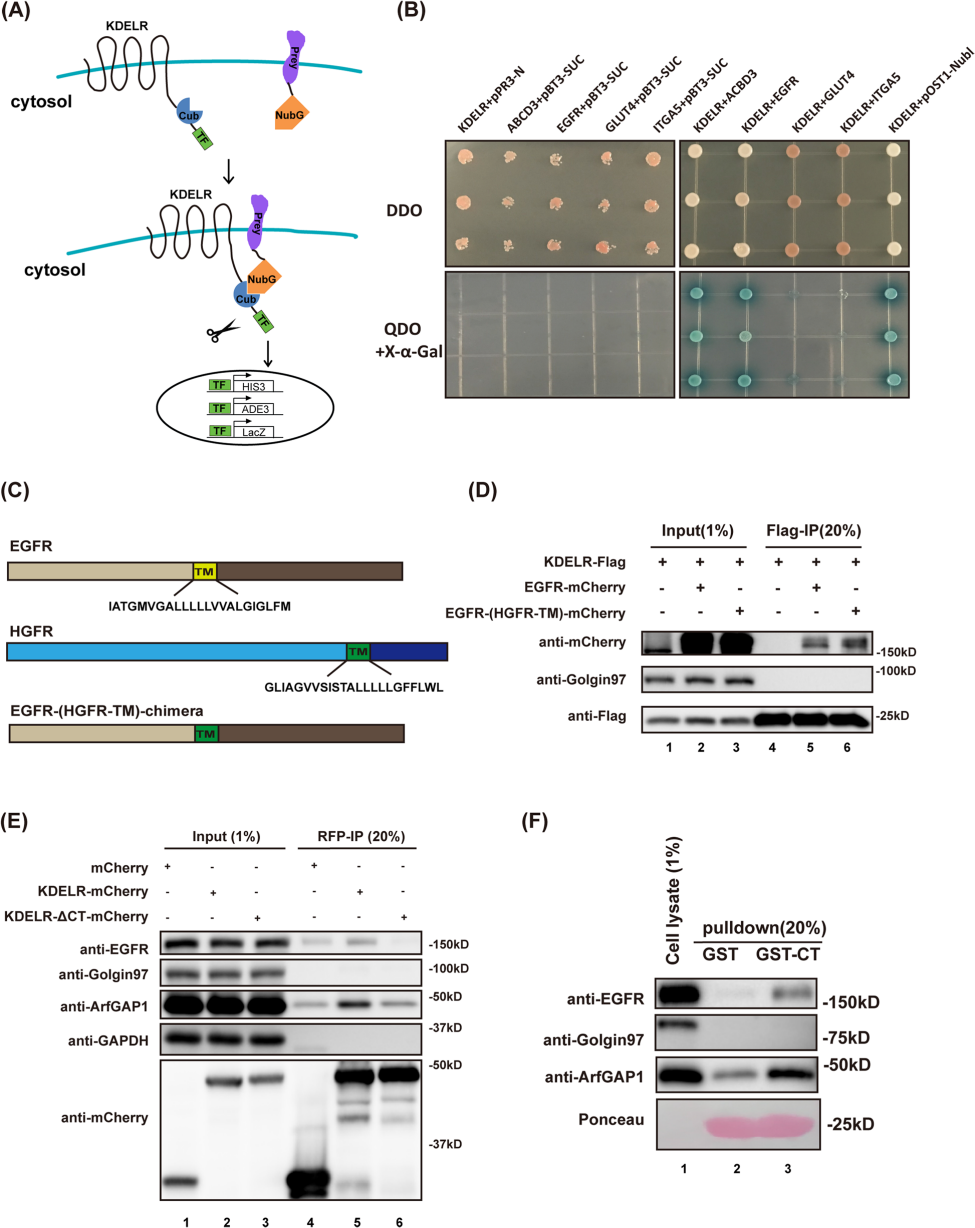
KDELR likely interacts with EGFR through its C-tail. **A** Schematic illustration of split-ubiquitin membrane yeast two hybrid (MYTH) assay. KDELR was fused to Cub and a transcription factor in the bait vector. ACBD3, EGFR, GLUT4, and ITGA5 were subcloned into the Nub-containing prey vector. Interaction between KDELR and a prey protein combines Cub and Nub to release the transcription factor and allow the transcription of HIS3, ADE3, and LacZ genes. **B** KDELR interacted with ACBD3 and EGFR, but not with GLUT4 and ITGA5, in MYTH assay. Yeast cells were transformed with indicated bait and prey plasmids and grown on transformation selection plates depleted of tryptophan and leucine (DDO, upper panel) or on interaction selection plates depleted of tryptophan, leucine, histidine, and adenine, plus 40 μg/ml X-α-Gal (QDO, bottom panel). **C** Schematic representation of EGFR chimera with EGFR transmembrane domain swapped for HGFR transmembrane motif. **D** EGFR chimera co-immunoprecipitated with KDELR-Flag. HeLa cells overexpressing KDELR-Flag and EGFR-mCherry or EGFR-(HGFR-TM)-mCherry were lysed and subjected to IP with anti-Flag sepharose, followed by immunoblotting analysis. **E** KDELR depleted of C-tail did not interact with EGFR. HeLa cells transfected with mCherry, KDELR-mCherry, or KDELR-ΔCT were processed for mCherry IP and western blotting. **F** The C-terminal tail of KDELR pulls down EGFR. Recombinant GST and GST-CT (KDELR-CT) proteins conjugated on glutathione beads were incubated with cell lysates prepared from A431 cells and subjected for immunoblotting with indicated antibodies

To confirm our finding in BiFC experiments, we used split-ubiquitin-based membrane yeast two-hybrid (MYTH) system. KDELR, the “bait” protein, was fused with the C-terminal fragment of ubiquitin (Cub) and a transcription factor. Acyl-CoA binding domain-containing protein 3 (ACBD3), EGFR, glucose transporter 4 (GLUT4), and integrin subunit α5 (ITGA5) were fused to a mutant of N-terminal fragment of ubiquitin (NubG), which carries an isoleucine to glycine point mutation to avoid the automatic association of Cub and Nub (Fig. 3A) [48]. ACBD3 was used as a positive control as it has been shown to bind KDELR directly [20]. Cell membrane receptors, GLUT4 and ITGA5, were included as negative controls.

The results from our MYTH assay indicated that co-expression of KDELR with ACBD3 or EGFR allowed yeast colonies to grow on synthetic dropout (SD) growth media that are supplemented with X-Gal, but do not contain Tryptophan, Leucine, Adenine, and Histine (Fig. 3B, QDO + X-Gal panel). Nubl, which binds Cub spontaneously, was used as a positive control in MYTH system. On the other hand, yeasts expressing KDELR with GLUT4 or ITGA5 grew on SD-Tryptophan-Leucine media (DDO) only (Fig. 3B). Co-expression of KDELR and pPR3-N prey vector, or prey proteins and pBT3-SUC bait vector was also contained as negative controls. Taken together, our data suggested that KDELR interacts with EGFR specifically.

### The C tail of KDELR interacts with EGFR

As a small protein equipped with seven transmembrane domains, KDELR has approximately 79% of its residues buried in the membrane. We first tested whether the interaction between EGFR and KDELR is mediated by their transmembrane regions. To this end, we swapped the transmembrane spans of EGFR and HGFR to make a chimeric protein (EGFR-(HGFR-TM)-mCherry) that is composed of the ectodomain and cytoplasmic region of EGFR, but the transmembrane motif of HGFR (Fig. 3C). HeLa cells co-expressing KDELR-Flag and EGFR-(HGFR-TM)-mCherry chimera or EGFR-mCherry, were analyzed in co-immunoprecipitation (co-IP) experiment using anti-Flag beads. The chimera protein was co-immunoprecipitated by KDELR as well as wildtype EGFR, suggesting that EGFR does not use its transmembrane motif to bind KDELR (Fig. 3D).

Since the C tail of KDELR is known to be important for its interaction with other proteins, we next asked whether it is required for EGFR binding. We transfected HeLa cells with mCherry, KDELR-mCherry, or KDELR-ΔCT-mCherry (C tail deleted) and immunoprecipitated mCherry tagged proteins with anti-RFP agarose. A significant amount of EGFR was found in the precipitate only in cells expressing wildtype KDELR. When C tail of KDELR was deleted, EGFR was no longer detected to be pulled down by KDELR-ΔCT-mCherry. ArfGAP1, which has been described to bind the C-terminus of KDELR, was also included as a positive control (Fig. 3E).

Then, we performed GST pulldown assays to further confirm the result of our co-IP experiment. GST and GST tagged KDELR C tail were purified from E. coli, immobilized onto glutathione beads, and incubated with cell lysates prepared from a epidermoid carcinoma cell line, A431, which has a high endogenous level of EGFR. EGFR as well as ArfGAP1 were pulled down by the C tail of KDELR efficiently, confirming that EGFR binds the C-terminus of KDELR (Fig. 3F) [49].

### EGFR mediates the endocytosis of KDELR

**Fig. 4:**
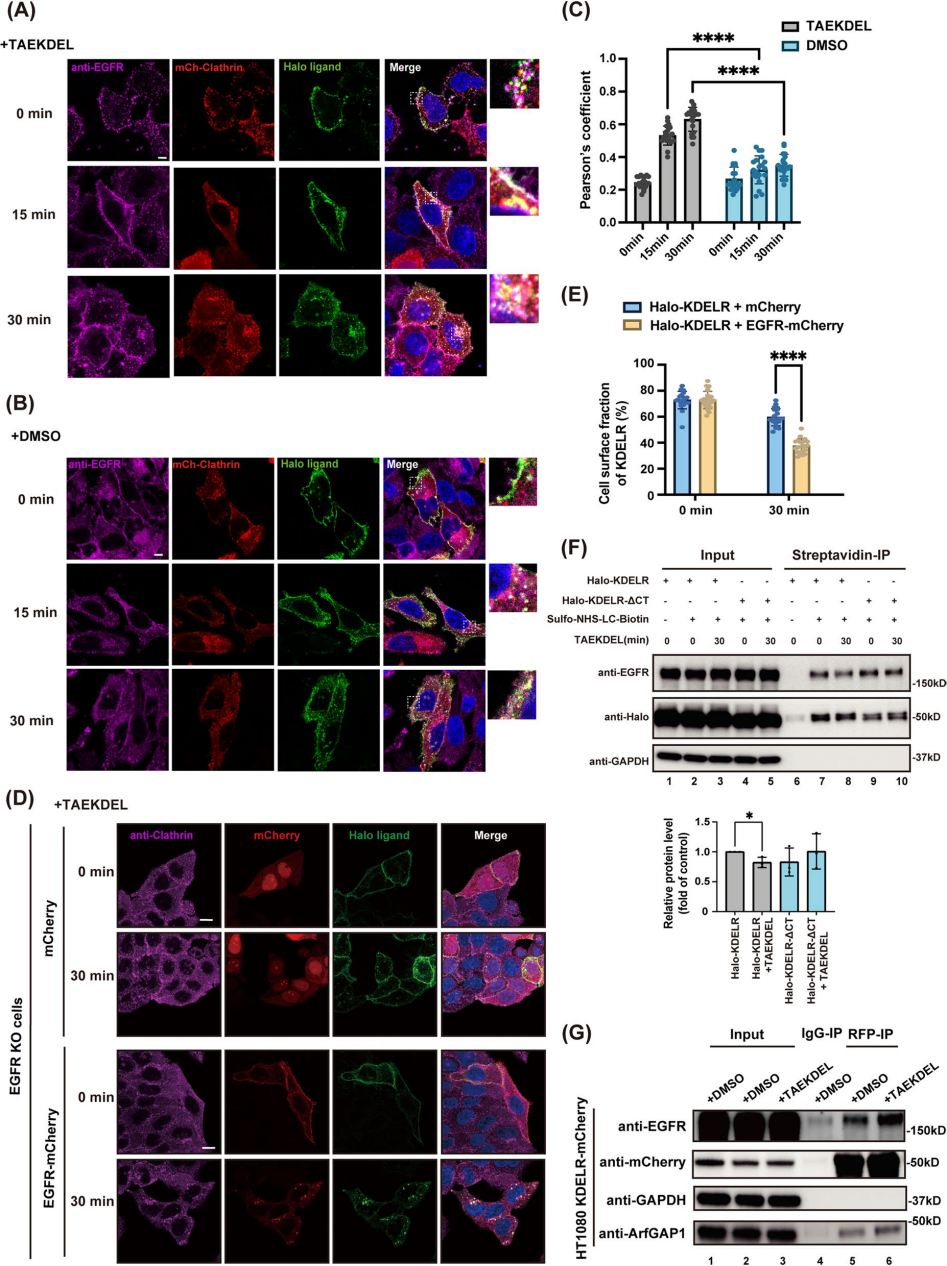
EGFR mediates the endocytosis of KDELR induced by KDEL ligand. **A-C** HeLa cells co-transfected with Halo-KDELR and mCherry-clathrin were stained with non-membrane permeable HaloTag Alexa Fluor 488 ligand and treated with 50 μM TAEKDEL peptide (**A**) or DMSO (**B**) at 4°C for 30 min, prior to incubation at 37°C for 0, 15, 30 min. Cells were stained with anti-EGFR antibody and observed by Zeiss LSM880 microscope. Regions of interest highlighted by dashed white lines were magnified. Scale bar = 10 μm. The co-localization of EGFR and KDELR in the Golgi area was represented by Pearson’s coefficient and analyzed using two-way ANOVA with a Sidak’s test (**C**). *n* = 20 cells pooled from 3 independent experiments. **D**,** E** The endocytosis of KDELR was impaired in EGFR-depleted cells. HeLa cells depleted of EGFR were transfected with Halo-KDELR and mCherry or EGFR-mCherry for 18 h and stained with non-membrane permeable HaloTag Alexa Fluor 488 ligand for surface-expressed KDELR. Cells were then treated with 50 μM TAEKDEL peptide at 4 °C for 30 min, prior to incubation at 37 °C for 0 or 30 min. Endogenous clathrin was stained with antibody, followed by confocal image analysis (**D**). The percentage of the surface green fluorescence signals in the total green signals was quantified and analyzed by two-way ANOVA with Sidak’s test (**E**). *n* = 20 cells pooled from 3 independent experiments. ****: *P* < 0.0001. Scale bars = 10 μm. **F** KDEL ligand did not induce the endocytosis of KDELR-ΔCT. HeLa cells depleted of KDELR were transfected with Halo-KDELR or Halo-KDELR-ΔCT and treated with 50 μM TAEKDEL peptide at 4 °C for 30 min. Cells were then incubated at 37 °C for 0 or 30 min, prior to labeling by sulfo-NHS-LC-Biotin at 4 °C for 10 min. Total surface proteins were pulled down by streptavidin beads and analyzed by immunoblotting. Relative protein levels on the cell surface were subjected to two-tailed, unpaired t tests (*n* = 3 independent experiments). *: *P* < 0.05. **G** TAEKDEL ligand incubation improved the binding between KDELR and EGFR. HT1080 stably overexpressing KDELR-mCherry cells were treated with 50 μM TAEKDEL peptide or DMSO at 4 °C for 30 min, prior to incubation at 37 °C for 30 min. Cell lysates were then prepared and subjected to RFP-IP analysis

Although we showed that EGFR is an interacting protein for KDELR so far, we have not demonstrated whether EGFR is the co-receptor on the cell surface that is responsible for mediating KDELR endocytosis. To selectively label surface-localized KDELR, we co-transfected HeLa cells with Halo-KDELR and mCherry-clathrin for 18 h, stained the cell membrane KDELR with non-membrane permeable fluorescent Halo ligands in living cells at 4°C, and then incubated cells with TAEKDEL peptide or DMSO for 0, 15, and 30 min at 37°C, prior to fixation and staining with anti-EGFR antibody. Confocal results showed that few surface-expressed Halo-KDELR co-localized with EGFR and clathrin at steady state. Upon addition of TAEKDEL ligand, co-localization of KDELR with EGFR and clathrin was improved dramatically over time, confirming that internalization of KDELR-EGFR complex undergoes clathrin-mediated endocytic pathway as reported before (Fig. 4A-B) [15]. A two-way ANOVA analysis indicated that co-localization of EGFR and KDELR was statistically significant only after TAEKDEL treatment, compared to DMSO controls (Fig. 4C).

In order to further confirm that EGFR mediates endocytosis of KDELR via CCV, we investigated internalization of KDELR in EGFR-depleted HeLa cells. As in wildtype HeLa cells, overexpressed Halo-KDELR was observed to localize to the plasma membrane in EGFR knockout cells. However, almost all of surface-labeled KDELR was retained on the plasma membrane after incubation with TAEKDEL peptide for 30 min, whereas more than 60% of KDELR were co-localized with EGFR and clathrin in endosomes and at the Golgi area in cells expressing EGFR-mCherry (Fig. 4D and E). Interestingly, a small fraction of KDELR was observed in endosomes close to the plasma membrane in EGFR-depleted cells, although the majority of KDELR remained on the cell surface (Fig. 4D).

As the C-tail of KDELR is seemingly responsible for EGFR binding, we also tested whether C tail-deleted KDELR is endocytosed upon KDEL ligand binding using cell surface biotinylation assay, as previously described [15]. HeLa cells stably knockdown of KDELR were transfected with Halo-KDELR or Halo-KDELR-ΔCT and incubated with KDEL ligand at 4°C for 30 min, prior to incubation at 37 °C for 0 or 30 min. All plasma membrane proteins were labeled by non-membrane permeable Sulfo-NHS-LC-Biotin, followed by streptavidin pulldown and western blot analysis. Incubation with TAEKDEL peptide induced significant reduction of surface-expressed EGFR and KDELR (Fig. 4F, lane 7&8). In contrast, in cells expressing Halo-KDELR-ΔCT, KDEL ligand did not change the surface fraction of either EGFR or KDELR-ΔCT, suggesting that KDEL ligand-induced internalization of surface-expressed KDELR requires its C-terminal tail (Fig. 4F, lane 9 &10).

### KDEL ligand binding improves the interaction between EGFR and KDELR

As we observed that co-localization of EGFR and KDELR was considerably enhanced by KDEL peptide incubation in confocal images, we next checked whether the binding of EGFR and KDELR increases after KDEL ligand addition. HT1080 cells stably overexpressing KDELR-mCherry were incubated with DMSO or TAEKDEL peptide for 30 min, prior to co-IP with anti-RFP agarose beads. As expected, addition of TAEKDEL peptide significantly increased EGFR precipitated by KDELR compared to DMSO treated samples (Fig. 4G), confirming that KDEL ligands may trigger the interaction between EGFR and KDELR.

We also observed that TAEKDEL ligand incubation induced a perinuclear localization pattern for clathrin, EGFR, and KDELR, whereas DMSO incubation did not (Fig. 4A and B). To further confirm whether this perinulcear localization is in the Golgi, we stained HeLa Halo-KDELR and KDERL knockdown cells with a specific antibody against a *trans*-Golgi network (TGN) marker, TGN46, after KDEL ligand or DMSO treatment. Indeed, a subset of EGFR was localized to the TGN after TAEKDEL incubation in a KDELR-dependent manner (Sup. Figure 4A-D).

To determine whether endocytosed EGFR induced by KDEL ligand undergoes lysosomal degradation, we incubated HeLa Halo-KDELR cells with 1.5 nM EGF (low concentration), 200 nM EGF (high concentration), TAEKDEL, or TAEAAAA peptides, respectively. As reported previously, a high dose of EGF treatment mostly led to lysosomal degradation of ~ 65% endocytosed EGFR, while a low dose of EGF resulted in much less degradation. Strikingly, KDEL ligand addition did not result in EGFR degradation at all (Sup. Figure 4E).

Taken together, our results suggested that EGFR seems to function as a co-receptor for KDELR on the plasma membrane, mediates its endocytosis via clathrin-coated vesicles upon KDEL-KDELR binding, leading to their Golgi-localization and cell surface-recycling of KDELR/EGFR, instead of degradation.

### Oligomerization of EGFR is moderately increased upon KDEL-KDELR binding

The canonical process of EGFR internalization starts with the binding of EGF with the extracellular domain of EGFR, followed by homo- or hetero-oligomerization of EGFR, followed by phosphorylation of its C-terminus. Activated EGFR exits the cell surface through clathrin-mediated endosomal pathway for subsequent recycling back to the cell surface or degradation by the lysosomal compartment [26].

**Fig. 5:**
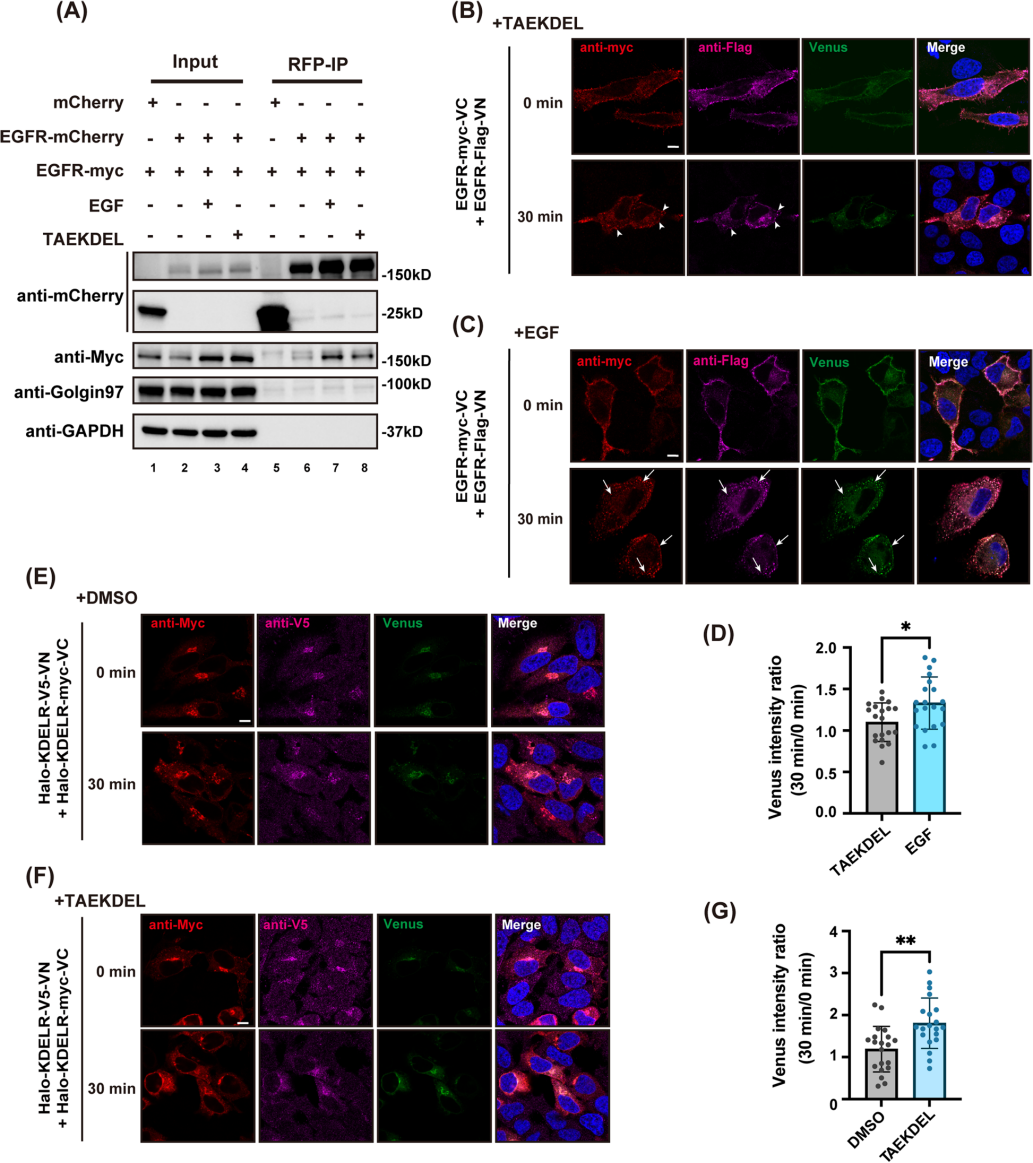
TAEKDEL peptide greatly increases KDELR oligomerization, but only moderately enhances EGFR oligomerization. **A** KDEL ligand induced moderate increase of EGFR oligomerization. HeLa cells co-expressing EGFR-myc with mCherry (negative control) or EGFR-mCherry were incubated with 30 nM EGF or 50 μM TAEKDEL peptide at 4 °C for 30 min. Cells were then processed for IP with mCherry and western blotting. **B-G** HeLa cells co-transfected with EGFR-myc-VC/EGFR-Flag-VN (**B**-**D**) or Halo-KDELR-V5-VN/Halo-KDELR-myc-VC (**E**–**G**) were treated with 50 μM TAEKDEL peptide or 30 nM EGF at 4 °C for 30 min, followed by incubation at 37 °C for 0, or 30 min. Cells were then fixed, stained with indicated antibodies, and observed under confocal microscope. Venus signal intensity ratio of 30-min incubation at 37°C versus 0-min was quantified using two-tailed t-test (**D** for results of **B** and **C**, **G** for results of **E** and **F**). *n* = 20 cells pooled from 3 independent experiments. *: *P* < 0.05. **: *P* < 0.01. Scale bars = 10 μm

First, we tested whether KDEL ligand-activated EGFR undergoes oligomerization as well. To this end, the interaction between two differently tagged EGFR monomers was examined by co-IP experiments. Cells were transiently transfected with EGFR-mCherry and EGFR-myc, followed by EGF or KDEL ligand incubation and IP of EGFR-mCherry using anti-RFP beads. As expected, EGFR-mCherry and EGFR-myc interacted with each other in the absence of ligand and their interaction was greatly enhanced by EGF incubation (Fig. 5A, lane 6&7). TAEKDEL peptide addition moderately increased co-IP of EGFR-mCherry and EGFR-myc, but to a much less extent than EGF addition (Fig. 5A, lane 6–8), suggesting that oligomerization of EGFR is moderately improved by KDEL binding to KDELR.

The oligomerization of EGFR was also evaluated by split Venus assay. HeLa cells overexpressing EGFR-myc-VC and EGFR-Flag-VN were incubated with TAEKDEL peptide or EGF for 0 and 30 min, prior to confocal analysis for Venus signals. As shown in Fig. 5B and D, EGFR oligomerization was observed even before TAEKDEL or EGF addition. KDEL ligand was not able to enhance the fluorescent signals in the Venus channel. In some endosome-like compartments, co-localization of EGFR-myc-VC and EGFR-Flag-VN in confocal images did not generate green signals, suggesting that EGFR monomers are in close proximity but are not close enough to bring the two halves of Venus together (white arrowheads in the bottom panels of Fig. 5B). In contrast, Venus signals were largely enhanced upon EGF stimulation especially in endosomes (white arrows in Fig. 5C), suggesting EGFR dimers appear in endosomal pathways after EGF binding, as previously described.

### KDELR oligomerization is greatly increased by KDEL ligand binding

Intracellular KDELR is known to self-oligomerize through its transmembrane domains [50]. On the cell surface, KDELR has been described to form clusters especially after cargo binding, suggesting that surface-expressed KDELR oligomerizes as well [12, 13]. To study oligomerization state of KDELR using split Venus assay, HeLa cells were co-transfected with Halo-KDELR-V5-VN and Halo-KDELR-myc-VC, followed by confocal microscope analysis for Venus signals. None of these two constructs had background venus signals when transfected individually in cells (Sup. Figure 3D and E). At steady state, weak Venus fluorescence was observed, suggesting that KDELR monomers oligomerize even when there is no ligand. The Venus signal increased almost twofold after 30 min of incubation with TAEKDEL peptide, whereas it remained unchanged with DMSO treatment (Fig. 5E-G). These results showed that KDEL binding to KDELR may facilitate the formation of KDELR dimers.

### The kinase activity of EGFR is required for the endocytosis of KDELR

**Fig. 6:**
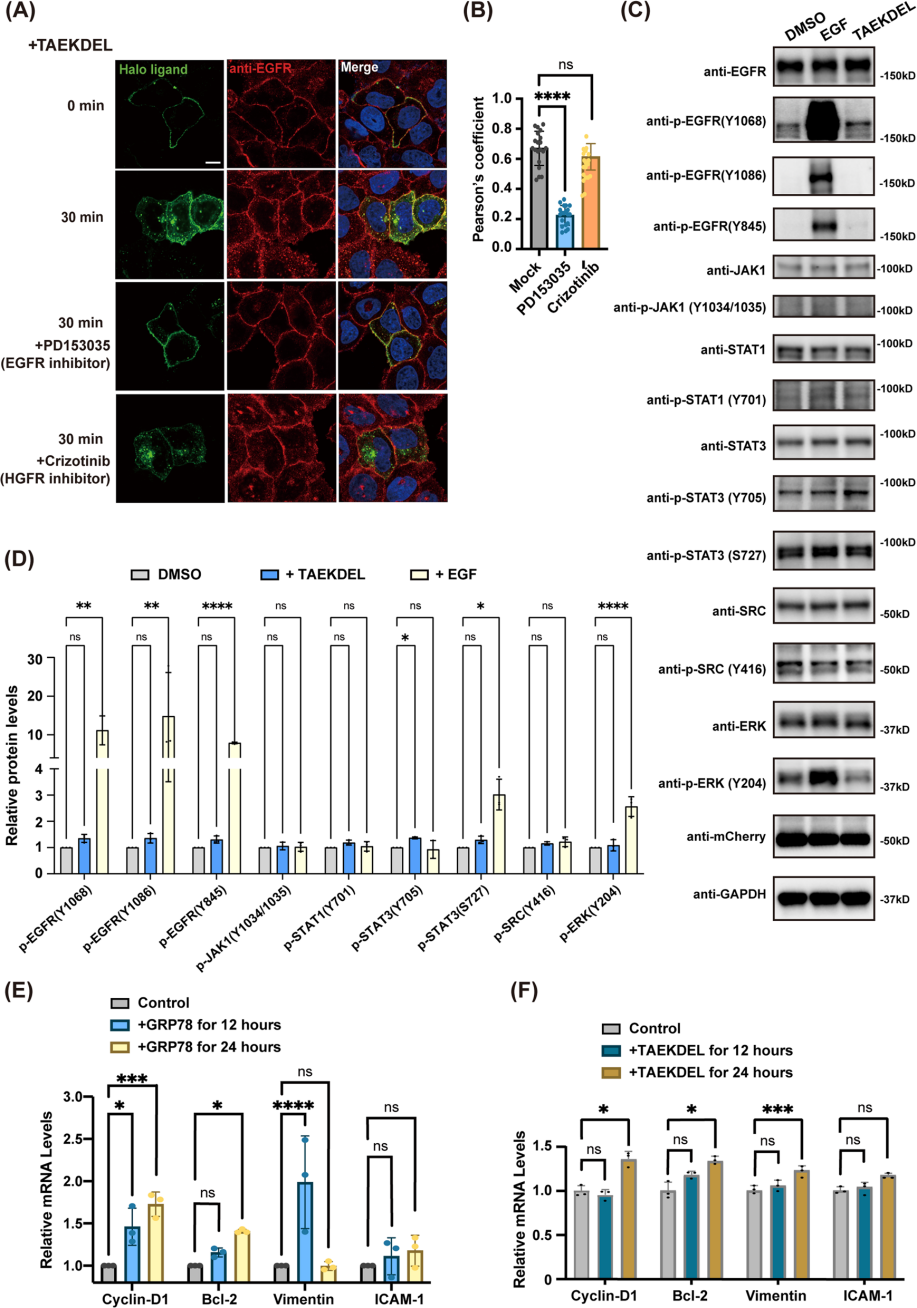
TAEKDEL peptide transactivates EGFR-STAT3 signaling pathway. **A**,** B** Endocytosis of KDELR induced by KDEL ligand was inhibited by a EGFR inhibitor. HeLa cells expressing Halo-KDELR were treated with non-permeable HaloTag Alexa Fluor 488 ligand and DMSO, 250 nM PD153035 or 20 nM Crizotinib at 37 °C for 2 hous, prior to incubation with 50 μM TAEKDEL peptide at 4 °C for 30 minitures. Cells were then incubated at 37 °C for 0 or 30 min and stained for endogenous EGFR (**A**). Co-colization of EGFR and KDELR in the peri-nuclear region was represented by Pearson’s coefficient and quantified using one-way ANOVA with a Dunnett’s multiple comparisons test (**B**). *n* = 20 cells pooled from 3 independent experiments. ****: *P* < 0.0001. Scale bar = 10 μm. **C**,** D** Phosphorylation of EGFR and STAT3 was increased by TAEKDEL incubation. HT1080 cells stably overexpressing KDELR-mCherry were incubated with DMSO, 1.5 nM EGF, or 50 μM TAEKDEL at 37 °C for 30 min. Cell lysates were prepared and subjected to immunoblotting. (**C**). Fold change of phosphorylated proteins was statistically analyzed using one-way ANOVA with Dunnett’s multiple comparisons (**D**). *n* = 3 independent experiments. *: *P* < 0.05. **: *P* < 0.01. ****: *P* < 0.0001. ns: not significant (**D**). **E**, **F** *Cyclin-D1, Bcl-2, Vimentin, ICAM-1* mRNA in HT1080 KDELR-mCherry cells treated with GRP78 (**D**) or TAEKDEL (**E**) at 37 °C was quantified using RT-PCR and analyzed by the ΔΔCT method. Statistical significance was calculated using two-way ANOVA with a Dunnett’s post-hoc test for multiple comparisons. *n* = 3 independent experiments. *: *P* < 0.05. ***: *P* < 0.001. ****: *P* < 0.0001. ns: not significant

Phosphorylation of tyrosine residues in trans in the carboxyl-tail of EGFR dimer is critical for the receptor’s internalization and downstream signaling. To investigate the role of EGFR phosphorylation in the endocytosis of KDELR, we transfected HeLa cells with Halo-KDELR overnight and added TAEKDEL peptide in the medium for 30 min in the presence of DMSO, EGFR kinase inhibitor (PD153035), and HGFR kinase inhibitor (Crizotinib). Strikingly, addition of PD153035 restrained surface-expressed EGFR and KDELR from endocytosis even after KDEL peptide treatment, whereas both receptors in DMSO and Crizontinib treated cells showed significantly increased localization in endosomes and at the Golgi upon TAEKDEL incubation (Fig. 6A and B). These results suggested that EGFR kinase inhibitor completely blocked the endocytosis of EGFR and KDELR specifically, as HGFR kinase inhibitor did not affect the internalization of EGFR and KDELR effectively.

### KDEL ligand binding to KDELR activates STAT3 signaling

EGFR and its interactors contribute to a complex network of signaling cascades, initiated by a variety of ligands. We tested activation of major EGFR signaling pathways with specific antibodies against phosphorylated proteins in western blotting experiment. HT1080 cells stably overexpressing KDELR-mCherry were incubated with DMSO, EGF or TAEKDEL peptides, prior to cell disruption and western blot analysis. As expected, EGF incubation greatly increased phosphorylation of EGFR (Tyr 1068, Tyr 1086, Tyr 845) and extracellular signal-regulated kinase (ERK), but not JAK, Src or STATs. In contrast, KDEL peptide incubation increased phosphorylation of STAT3 (Tyr705, but not Ser727) by ~ 37% compared to the control cells, whereas JAK, Src, and ERK kinases were not activated, indicating that STAT3 activity may be specifically increased after TAEKDEL binding to KDELR on the cell surface (Fig. 6C and D).

As a transcription factor, activated STAT3 has been described to initiate cell proliferation and migration by translocating to the nucleus and turning on transcription of target genes [51, 52]. To assess the function of activated STAT3 by KDEL ligand, we selected four STAT3-regulated genes known to be involved in cell proliferation/migration and investigated their transcription by reverse transcription- polymerase chain reaction (RT-PCR) assay. Total mRNA samples isolated from HT1080 cells were reverse transcribed and quantified by real-time PCR. As shown in Fig. 6E and F, mRNA levels for *Cyclin-D1* and *Bcl-2* gene, which induce cell proliferation and inhibit apoptosis, were upregulated in GRP78- or TAEKDEL-incubated cells. Two genes that promote cell migration were differently affected by GRP78 treatment. Transcription of *Vimentin* gene was greatly increased by GRP78 or TAEKEL incubation, while *ICAM-1* gene transcription was not significantly changed.

### Inhibitor of STAT3 suppresses cell proliferation and migration activated by KDEL ligand

To further dissect the function of STAT3 signaling activated by KDEL peptide, we first investigated whether STAT3 activation was necessary for the endocytosis of KDELR. Cells expressing Halo-KDELR were incubated with TAEKDEL peptide in the presence of DMSO or cryptotanshinone, a STAT3 inhibitor, for 30 min, followed by staining with anti-GM130, a Golgi marker, and anti-clathrin antibodies. Addition of KDEL ligand increased co-localization of KDELR with GM130 and clathrin, which was not changed by cryptotanshinone incubation, indicating that STAT3 is unlikely to be involved in the endocytosis of KDELR (Sup. Figure 5A and B).

**Fig. 7:**
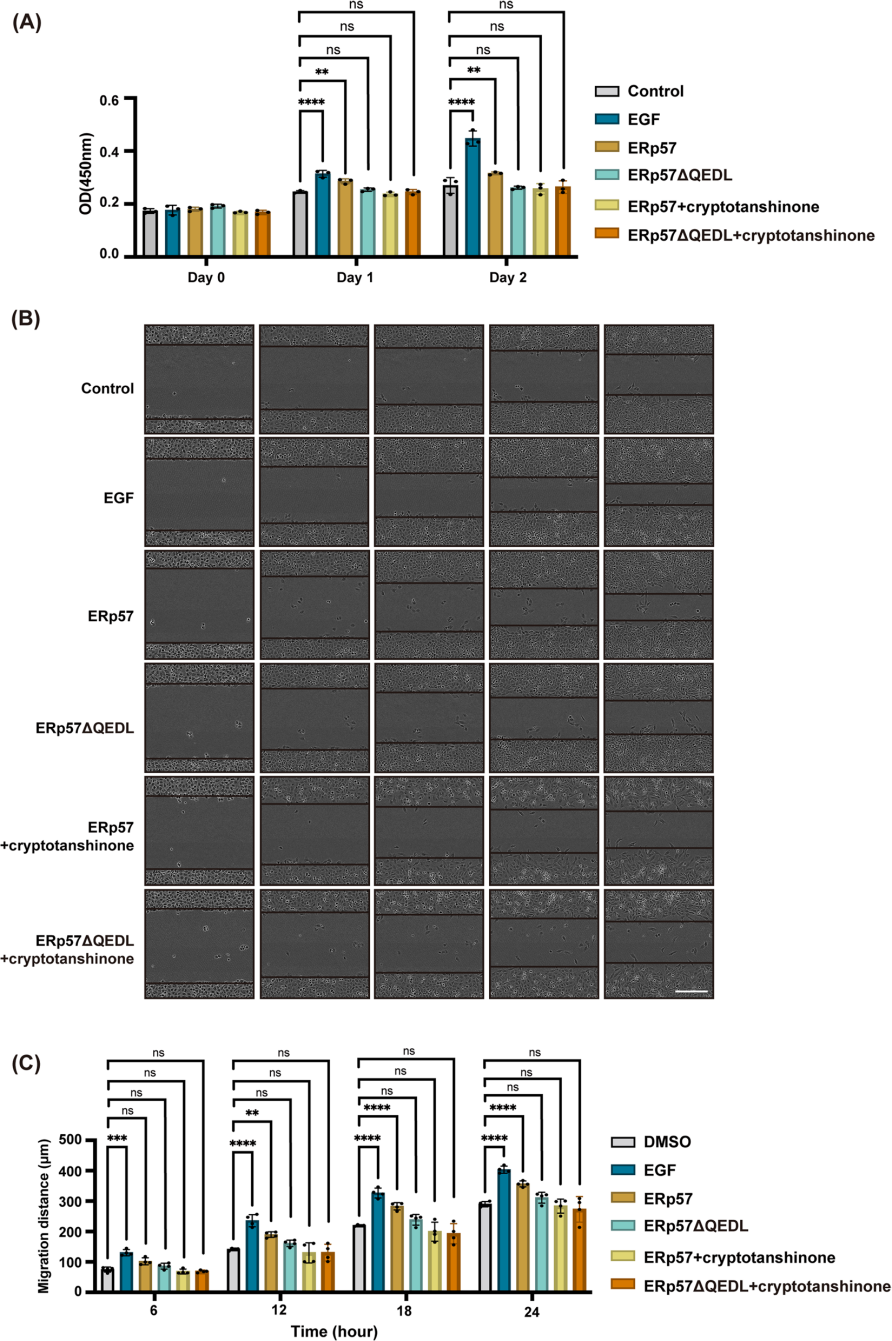
STAT3 inhibitor attenuates cell proliferation and migration induced by ERp57. **A** STAT inhibitor abrogated cell proliferation induced by ERp57. Cell growth of HT1080 cells was measured by CCK-8 assay on day 0, 1, and 2 after incubation with 30 nM EGF, 50 nM ERp57 or ERp57ΔQEDL, in the absence or presence of 4 μM cryptotanshinone, in MEM supplemented with 10% FBS. OD450 measurements of cells at indicated time points were represented by histogram. Statistical analysis was performed using two-way ANOVA with a Dunnett’s post-hoc test for multiple comparisons. *n* = 3 independent experiments. **: *P* < 0.01.****: *P* < 0.0001. **B**, **C** STAT3 inhibitor suppressed cell migration in wound healing assay. Phase contrast images of HT1080 cells incubated with indicated reagents at different time points after wounding (**B**). The migration distance in wound healing was statistically analyzed using two-way ANOVA with a Dunnett’s post-hoc test for multiple comparisons (**C**). *n* = 4 independent experiments. **: *P* < 0.01. ***: *P* < 0.001.****: *P* < 0.0001. ns: not significant. Scale bar = 300 μm

Next, we asked whether STAT3 signaling is responsible for ER proteins-induced cell proliferation using CCK-8 assay. The growth rate of HT1080 cells seeded on a 96-well plate was evaluated by OD450 measurements on 0, 1, and 2 days, in growth medium supplemented with ERp57 or ERp57ΔQEDL with or without cryptotanshinone, a chemical that strongly inhibits phosphorylation of Tyr705 on STAT3. Similar to what we observed with GRP78 and MANF, ERp57 induced cells to grow at a rate significantly higher than control and ERp57ΔQEDL-treated cells on both day 1 and day 2. However, ERp57/cryptotanshinone incubated cells revealed similar proliferation rate as control cells (Fig. 7A). These results suggested that ERp57-induced cell proliferation is controlled by STAT3 signaling.

Impact of cryptotanshinone was also evaluated using the wound healing assay. Confluent HT1080 cells were scratched and grown in medium supplemented with EGF, purified ERp57 or ERp57ΔQEDL in the absence or presence of cryptotanshinone. Cells incubated with ERp57 only were observed to heal faster than control cells at all time points tested after a scratch. In contrast, cells treated with ERp57 and cryptotanshinone showed about the same healing speed as control cells, suggesting that STAT3 inhibitor completely abrogated the effect of ERp57 on cell migration (Fig. 7B and C).

## Discussion

**Fig. 8:**
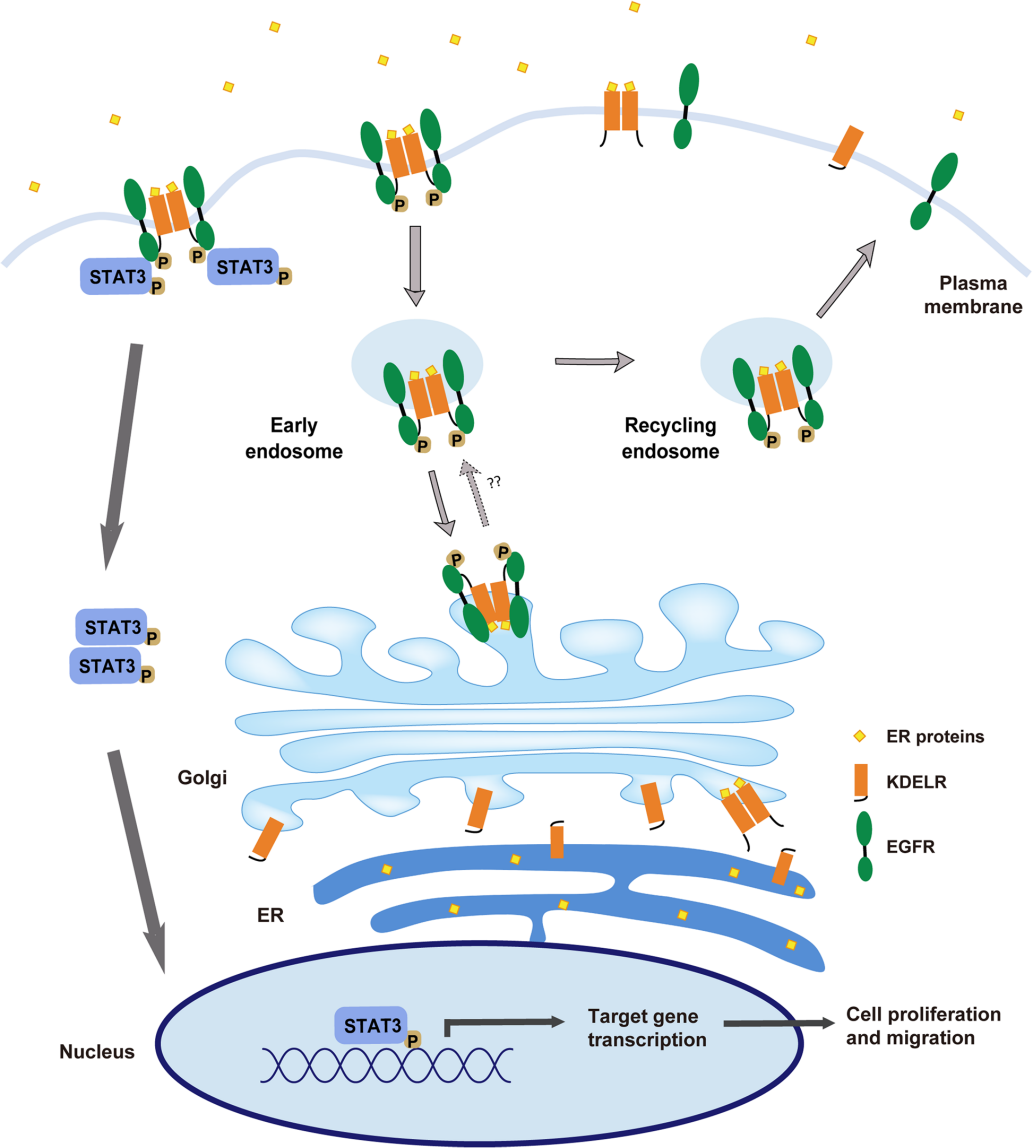
A model of EGFR-mediated KDELR endocytosis and STAT3 signaling activation induced by KDEL ligand. Secreted ER proteins bind to surface-localized KDELR and induce oligomerization of the receptor, leading to KDELR-interacting EGFR monomers in close proximity for tyrosine phosphorylation. Phosphorylated EGFR initiates clathrin-mediated internalization of KDELR and EGFR, resulting in transport of these two receptors to the Golgi apparatus and back to the cell surface. STAT3 activated by phosphorylated EGFR transmits signals to the nucleus and induces transcription of genes that control cell proliferation and migration

In this study, we provide several evidences that surface-expressed KDELR oligomerizes upon KDEL ligand binding and undergoes accelerated endocytosis through its interaction with EGFR. Internalized KDELR and EGFR are then transported to the Golgi via clathrin-mediated vesicles and recycled back to the plasma membrane. Moreover, KDEL ligand binding induces phosphorylation of C-terminal tyrosine residues on EGFR, resulting in the phosphorylation of STAT3. Activated STAT3 then translocates to the nucleus and activates transcription of various target genes to promote cell proliferation and migration (Fig. 8).

C-terminal KDEL tetrapeptides are the retrieval signal for ER soluble proteins. However, this mechanism based on the binding of KDELR and KDEL-containing proteins is leaky. Proteins fused to the C-terminal retention motifs of ER proteins are found not only in the ER or at the Golgi, but also secreted to the extracellular space, especially under ER stress [53–55]. In fact, more ER proteins are secreted in cancer cells than in healthy cells due to the elevated stress conditions that are frequently encountered during cancer progression [6, 56]. Interestingly, the interaction between KDELR and its KDEL-bearing ligands is pH-dependent with an optimal high-affinity binding occurring in the range of pH 5.0 to 5.5, which matches the mildly acidic microenvironment of cancer cells favored for cancer development [36, 57, 58].

Our previous results have demonstrated that KDEL peptides accelerate the endocytic internalization of KDELR via endosomal transport [15]. Here we extended these findings by showing that the endocytosis of KDELR is mediated by its binding to EGFR through its C-tail, which is highly improved upon KDEL ligand addition. The crystal structural data of KDELR reveal that the carboxyl terminus of KDELR is buried inside the apo receptor. KDEL ligand binding induces a conformational change in the preceding transmembrane domain of the C-tail, which allows the C-terminal residues to be exposed for potential interaction with other proteins or receptors [36]. Indeed, our data from split Venus experiments clearly showed increased interaction between KDELR and EGFR after KDEL ligand incubation (Fig. 2D and E). Recent studies have shown that C-terminal tags may interfere with KDELR's functions. Due to lack of good commercial antibodies against endogenous KDELR, we were not able to validate our results using untagged endogenous KDELR. However, most of the studies on KDELR have used C-terminally tagged constructs, which were able to bind ArfGAP1 and recruit ARF1 for Golgi-to-ER traffic, suggesting that the interactions between C-terminally tagged KDELR and its binding partners are intact [50]. We also observed that KDELR is internalized even in EGFR-depleted cells, although less endocytic puncta of KDELR were found compared to wildtype cells (Fig. 4D). It is possible that KDELR has other co-receptors on the plasma membrane, although with lower affinity than EGFR. It is also likely that KDELR undergoes internalization via non-clathrin pathways, such as caveolae/lipid raft-dependent endocytosis, in the absence of EGFR. We have previously observed that caveolin co-localizes with wildtype KDELR, but to a much lower extent than the clathrin-dependent pathway, suggesting that potential caveolin-mediated KDELR endocytosis may be ligand-independent [15].

Auto-phosphorylation of the carboxyl tail in EGFR dimers is a critical step for the internalization of the receptor. The structure of EGFR monomers demonstrates that intramolecular interaction in the ectodomain of the receptor inhibits its oligomerization by occluding the dimerizing surface. EGF binding to EGFR induces conformational changes to expose the oligomerization arms, resulting in the formation of EGFR dimers and subsequent phosphorylation on tyrosine residues [24, 59]. However, there is controversy over whether oligomerization of EGFR is required for the endocytosis of the receptor. The recruitment of the Casitas b-lineage lymphoma (Cbl) by growth factor receptor-bound protein 2 (Grb2) to EGFR has been shown to be sufficient for the internalization of EGFR [60]. On the other hand, oligomerization of EGFR is proposed to be necessary for bringing the endocytic motifs on two monomers in close proximity for internalization [61]. Our results indicate that before ligand binding, both EGFR and KDELR spontaneously oligomerize and interact with each other at a low level. KDEL ligand binding to KDELR greatly improves oligomerization of KDELR, but only moderately enhances EGFR oligomerization, which is sufficient to activate the endocytosis of both receptors.

EGF triggered endocytosis of surface EGFR leads to intracellular EGFR accumulation in endosomes for recycling back to the plasma membrane or degradation in the lysosomes. KDEL ligand incubation seems to prefer the recycling itinerary as no degradation of EGFR was detected in our results. In fact, a significant subset of internalized EGFR and KDELR were localized to the Golgi after 30 min of treatment with KDEL peptides. It is possible that the Golgi-fraction KDELR may activate the COPI-dependent retrograde trafficking pathway to be further transported to the ER. Golgi-localized EGFR may be activated by its ligands, although it requires further in-depth investigation.

In addition to inducing endocytosis, EGFR activation also initiates signaling cascades, mainly including Ras/mitogen-activated protein kinase (MAPK) pathway, phosphatidylinositol-3-kinase (PI3K) pathway, phospholipase C-gamma (PLCγ) pathway, and STAT3 pathway. Protein effectors in these downstream signaling pathways are recruited to the cytoplasmic phosphorylated tyrosine docking sites on EGFR primarily through Src homology 2 (SH2) and phosphotyrosine binding (PTB) domains. STAT3 utilizes its SH2 domain to interact with phosphorylated EGFR, leading to subsequent phosphorylation of STAT3 by JAK, Src kinase, or active EGFR. Then, reciprocal phosphotyrosine and SH2 domain interactions enable oligomerization of STAT3 and translocation to the nucleus for regulation of target gene transcription [28, 62]. KDEL ligand activated EGFR displays moderate, but significant phosphorylation of tyrosine 1068, which is able to recruit and activate downstream effectors and kinases, such as Grb2 and STAT3. Interestingly, Grb2, which is a key adaptor protein for the activation of ERK, competes with STAT3 for phosphotyrosine residues on EGFR and down-regulates phosphorylation of STAT3 [63]. Our results indicated that only STAT3, but not ERK, Src or JAK, was phosphorylated upon TAEKDEL peptide incubation, suggesting that the activation of STAT3 by KDEL ligand is likely due to the phosphorylated EGFR.

Several signaling pathways have been proposed that may mediate the effects of secreted ER chaperones. Cell surface GRP78 was reported to facilitate cell proliferation and migration by activating PI3K and MAPK pathways in hepatoma cells, blocking TGF-β signaling in HeLa cells, and inducing STAT3 signaling in hepatocellular carcinoma cells [4, 64, 65]. ERp57 was demonstrated to associate with STAT3 in nucleus and regulate STAT3 activity in radioresistant laryngeal cancer cells [66]. Our data indicated that secreted ER chaperones, despite their variable sequences and functions, may utilize their common receptor, KDELR, on the plasma membrane to enhance cell growth and migration by activating EGFR-STAT3 pathway. It is possible that individual ER chaperone may have additional receptor(s) and subsequent downstream signaling pathway(s) in addition to the KDELR-EGFR-STAT3 pathway.

The conventional EGFR signaling is activated by an EGF-like ligand. Recent studies have discovered that EGFR may be activated by other cell surface receptors, such as GPCRs. Two major mechanisms of EGFR transactivation by GPCR have been proposed: GPCR-mediated release of EGFR ligands and the intracellular signaling crosstalk [67]. However, unlike these two well characterized mechanisms, transactivation of EGFR by KDELR is likely mediated by a direct interaction between the cytoplasmic regions of these two receptors. Upon KDEL binding, KDELR adopts a conformation that allows preferable binding to itself and to EGFR, most likely leading to formation of a heterotetramer with a dimer of KDELR and two EGFR molecules and subsequent phosphorylation of EGFR necessary for downstream signaling activation.

Overall, the current study describes the molecular mechanism of KDEL ligand-mediated, EGFR-dependent endocytosis and signaling of KDELR. This modified KDELR-EGFR crosstalk-dependent signaling reveals a previously unreported system of regulation for cell proliferation and migration.

## Material and methods

### Reagents and antibodies

The following antibodies were used: anti-GAPDH (HRP-60004, Proteintech), anti-EGFR (4267S, Cell Signaling Technology), anti-Myc (2278S, Cell Signaling Technology), anti-Golgin97 (13,192, Cell Signaling Technology), anti-mCherry (ab167453, Abcam), anti-Clathrin heavy chain (ab21679, Abcam), anti-Transferrin Receptor (ab214039, Abcam), anti-β-COP (ab2899, Abcam), anti-Halo (G9211, Promega), anti-AP1M1 (12,112–1-AP, Proteintech), anti-E-Cadherin (20,874–1-AP, Proteintech), anti-ARFGAP1(ab204405, Abcam), anti-Flag (F1804-1MG, Sigma), anti-TGN46 (AHP500GT, Bio-Rad), anti-p-EGFR (Tyr1068) (3777S, Cell Signaling Technology), anti-p-EGFR (Tyr1086)(ab32086, Abcam), anti-p-EGFR (Tyr845) (sc-57542, Santa Cruz Biotechnology), anti-STAT1 (9172, Cell Signaling Technology), anti-p-STAT1 (Tyr701) (9167, Cell Signaling Technology), anti-STAT3 (ab68153, Abcam), anti-p -STAT3 (Tyr705) (ab76315, Abcam), anti-p-STAT3 (Ser727) (ab76315, Abcam), anti-ERK (4695, Cell Signaling Technology), anti-p-ERK (MA515174, Invitrogen), anti-JAK1 (66,466–1, Proteintech), anti-p-JAK1 (Y1034/1035) (66245S, Cell Signaling Technology), anti-Src (2109S, Cell Signaling Technology), anti-p-Src Family (Tyr416) (6943S, Cell Signaling Technology), HaloTag Alexa Fluor 488 Ligand (G1002, Promega), HaloTag TMR Direct™ Ligand (G2991, Promega). Anti-Rabbit Alexa Fluor 488 (A21441), Alexa Fluor 568 (A10042), Alexa Fluor 647 (A21245), anti-Mouse Alexa Fluor 488 (A21200), Alexa Fluor 568 (A10037), and Alexa Fluor 647 (A21236) for immunofluorescence (IF) were obtained from ThermoFisher. Lentivirus concentration solution (AC04L442) was purchase from Life iLab Bio. TAEKDEL and TAEAAAA peptides were synthesized by GenScript.

### Cell culture, transfection and lentivirus infection

HeLa cells (ATCC, CCL-2) were grown in Delbecco’s modified Eagle medium (DMEM, Meilunbio) supplemented with 10% fetal bovine serum (FBS, ExCell Bio). HT-1080 cells (Stem Cell Bank, Chinese Academy of Sciences) were grown in Minimum Essential Medium (MEM, Meilunbio) with 10% fetal bovine serum (FBS, ExCell Bio). Transfection of plasmids was performed using Lipofectamine 2000 (11,668,019, Thermo-Fisher), according to the manufacturer’s instructions. For gene overexpression experiments, cells were transfected for 18 h, prior to co-IP and IF experiments.

To stably knock down KDELR in HT1080 cell line, the lentivirus expressing KDELR-shRNA (GGTTGCCAAACACTAAATCTG, targeting 3′-UTR) was packaged and commercially provided by Shanghai GenePharma, China. Cells were infected with the lentivirus using polybrene overnight. Three days after infection, the cells were cultured in puromycin (0.6 μg/ml) for five days.

HT1080 cells stably overexpressing KDELR-mCherry or Halo-KDELR were prepared using lentivirus expressing KDELR-mCherry or Halo-KDELR respectively. To produce and pack lentivirus expressing KDELR-mCherry, a lentiviral vector containing KDELR-mCherry along with packing (psPAX2) and envelop (pMD2.G) vectors were transfected into 293FT cells using Lipofectamine 2000. The supernatants were collected after transfection for 48 h and 72 h, filtrated with 0.45 nm filters, and concentrated using a Lenti-concentration kit (AC04L441, life-ilab). The lentivirus expressing Halo-KDELR was packaged and commercially provided by OBiO Technology.

### Scratch wound healing assay

Cells were seeded into a 96-well (Corning) and gown to full confluency in MEM supplemented with 10% FBS. Then cells were scratched using a wound maker (IncuCyte) to create homogeneous, 700–800 μm wide wounds in cell monolayers. After wounding, cells were washed twice with sterile PBS and incubated in MEM supplemented with 0.3% FBS and EGF, or recombinant ER proteins for 24 h. During this time period, real-time gap distances were imaged and determined using a high-throughput screening system (IncuCyte ZOOM).

### Reverse transcription and quantitative real time (qPCR) Assay

Total mRNA extraction was performed using using RNA Isolation kit (R0026, Beyotime). The reverse transcription was carried out by HiScript II Q RT SuperMix (R223-01, Vazyme) according to manufacturer’s protocol. Real time-PCR was performed by a QuantStudio 7 real-time PCR system (ThermoFisher) using ChamQTM Universal SYBR qPCR Master Mix (Q711-02, Vazyme). *GAPDH* mRNA was used for normalization. Primers are listed as follows: *KDELR* forward: AGCCACTACTTGTTTGCGCTA, *KDELR* reverse: CCTGCCACAATGGCGATGA; *Cyclin-D1* forward: GCTGCGAAGTGGAAACCATC, *Cyclin-D1* reverse: CCTCCTTCTGCACACATTTGAA; *Bcl-2* forward: GGTGGGGTCATGTGTGTGG, *Bcl-2* reverse: CGGTTCAGGTACTCAGTCATCC; *Vimentin* forward: GACGCCATCAACACCGAGTT, *Vimentin* reverse: CTTTGTCGTTGGTTAGCTGGT; *ICAM-1* forward: ATGCCCAGACATCTGTGTCC, *ICAM-1* reverse: GGGGTCTCTATGCCCAACAA; *GAPDH* forward: ACCACAGTCCATGCCATCAC, *GAPDH* reverse: TCCACCACCCTGTTGCTGTA.

### CCK-8 assay

Cell viability was analyzed by Cell Counting Kit-8 (Meilunbio) according to the manufacturer’s protocols. Cells were seeded at a density of 0.2 × 10^4^/well or 1.0 × 10^4^/well into a 96-well plate for being cultured in medium supplemented with 10% FBS or with 0.1% FBS, respectively. At 0, 24, and 48-h time points, 10% volume of CCK-8 reagent was added to the medium and incubated for 1 h. The absorbance was recorded at 450 nm using a microplate reader (ThermoFisher Scientific).

### Mass spec-based proteomics for cell surface proteins

HeLa cells (three 10 cm dish of cells for each experimental condition) were transfected with Halo-KDELR for 18 h and incubated with DMEM medium supplemented with 10% FBS and non-membrane permeable HaloTag PEG-biotin ligand (Promega) or DMSO for 30 min at 4°C. After incubation, the cells were washed three times with cold PBS, and harvested in 1 mL lysis buffer (20 mM HEPES, pH 7.4, 100 mM NaCl, 2 mM DDM (N-Dodecyl-β-D-Maltoside, Anatrace), 0.02% CHS (Cholesteryl hemisuccinate, Sigma), protease inhibitors (Bimake)). Then the cells were lysed by passing through a 1 mL syringe needle for 15 times and incubated a 4°C for 1 h. The lysates were cleared by centrifugation at 15,000 g for 20 min. The supernatants were incubated with Streptavidin beads for 1 h at 4 ^o^C. The beads were washed three times with cold PBS, and the proteins were eluted by boiling in Laemmli SDS sample buffer and subjected to SDS-PAGE for mass spectrometry. Samples were prepared by in-gel digestion, separated and analyzed on an Easy-nLC 1000 liquid chromatograph coupled to a Q Exactive HF mass spectrometer (ThermoFisher Scientific).

### Co-IP and immunoblotting

For co-IP experiments, total lysates were prepared using lysis buffer (20 mM HEPES, pH 7.4, 100 mM NaCl, 2 mM DDM (n-dodecyl-β-D-maltoside, Anatrace), 0.02% CHS (cholesterol hemisuccinate, Sigma), protease inhibitors (Roche)). Then, the lysates were passed through a syringe needle for 15 times and incubated with agitation for 1 h at 4°C. The supernatants were prepared by centrifugation at 15,000 g for 10 min and incubated with anti-RFP agarose beads (MBL life science) or anti-Flag agarose beads (GenScript) for 4 h at 4 ^o^C. The beads were washed three times with cold PBS. The protein were boiled in 2 × SDS running buffer and subjected to SDS-PAGE for western blotting.

For immunoblotting, proteins were separated by SDS-PAGE (Gensript) and transferred to the nitrocellulose membranes (Amersham). Then, the membranes was blocked for 1 h at room temperature with bovine serum albumin (BSA) in the blocking buffer (ABCONE), probed with specific primary antibodies overnight at 4 ^o^C, and incubated with peroxidase-conjugated secondary antibodies (Jackson Immuno Research). The bands were visualized with chemiluminescence (Clarity Western ECL Substrate, Bio-Rad) and imaged by a ChemiDoc Touch imaging system (Bio-Rad).

### IF staining and confocal microscopy

Cells grown on a 24-well glass bottom plate (Cellvis) were fixed with 4% paraformaldehyde (PFA) for 10 min, permeabilizated in permeabilization buffer (0.05% Triton-X100 in PBS) for 3 min, and blocked in blocking buffer (3% BSA, 0.05% Triton-X100 in PBS) for 60 min. Then the cells were incubated with primary and secondary antibodies in blocking buffer for 1 h. The nucleus was stained with Hoechst-33342 Santa cruz Biotechnology). Cells were washed three times with wash buffer (0.2% BSA, 0.05% Triton-X100 in PBS) and twice with PBS. Images were acquired using a Zeiss LSM880 confocal microscope with a 63 × oil immersion objective.

### Split-ubiquitin membrane yeast two hybrid assay

Human KDELR cDNA was subcloned in frame and upstream of the C-terminal half of ubiquitin (Cub) and the artificial transcription factor LexA-VP16 in the pBT3-SUC bait vector. ACBD3, EGFR, GLUT4, and ITGA5 cDNA were individually fused to the mutated N-terminal half of ubiquitin (NubG) in the pPR3-N prey vector. The NMY51 yeast strain co-transformed with bait and prey pair was spread onto selective medium lacking leucine and tryptophan (SD/-Leu/-Trp, DDO, Clontech). The physical binding of bait and prey was identified by colony selection in selective medium lacking adenine, leucine, tryptophan, and histidine (SD/-Ade/-Leu/-Trp/-His, Clontech) supplemented with X-α-Gal. Co-transformation of KDELR-Cub and pOST1-NubI was performed as a positive control.

### GST-pulldown assay

KDELR C-terminal (CT, residues 205–212) was inserted in pGEX-6p-1 plasmid for the expression of GST fusion proteins. The constructed plasmids were transformed into BL21 (DE3) (Vazyme) competent cells. Single colony of cells was grown in LB broth at 37°C until OD600 reading was between 0.6 and 0.8. Then proteins expression was induced by addition of 0.3 mM IPTG (Isopropyl β-d-thiogalactopyranoside) at 37°C for 3 h. Subsequently, bacteria were collected in lysis buffer (50 mM Tris–HCl, pH 7.4, 100 mM NaCl, 1 mM dithiothreitol 0.5% Triton X-100) and lysed by sonication. The supernatants were collected by centrifugation at 15,000 × g for 10 min, and incubated with Glutathione HiCap Matrix (Qiagen) at 4°C for 1h. The beads were washed by lysis buffer and the GST fusion proteins were eluted by 10 mM glutathione in lysis buffer.

For the pulldown assay, 100 ug of each GST-fusion protein was immobilized on glutathione Sepharose 4B (GE Healthcare). The resins were washed 3 times with lysis buffer and incubated with HeLa or HT1080 cell lysates at 4°C for 1 h. The resins were washed three times, resuspended in 2 × SDS loading buffer, and subjected to SDS-PAGE analysis.

### Image processing and statistical analysis

Pearson coefficient was analyzed by Fiji software. Statistical analyses were performed with GraphPad Prism 9.0 software using student’s t-test or ANOVA and represented as mean ± SD. *n* is noted in the figure legends.

### Supplementary Information

**Additional file 1: Supplementary Figure 1.** KDEL ligands induce cell growth and migration. A Recombinant His-tagged GRP78 and GRP78ΔKDEL were purified from E. coli and analyzed by Coomassie staining. B TAEKDEL peptide induced cell proliferation. HeLa cells were incubated with DMSO, 10 nM EGF, 50 μM TAEKDEL in DMEM supplemented with 0.1% FBS and subjected to CCK-8 assay on day 0, 1, and 2. Statistical analysis was performed using two-way ANOVA with a Dunnett’s post-hoc test for multiple comparisons. *n*=3 independent experiments. ns: not significant. **: *P*<0.01. C His-tagged MANF and MANFΔRTDL were purified from E. coli and analyzed by Coomassie staining. D MANF stimulates cell proliferation. Cell viability was measured by CCK-8 assay on day 0, 1, and 2 after incubation with 30 nM EGF, 50 nM MANF, or 50 nM MANFΔRTDL. Histogram summarized the OD450 measurements of cells at indicated time points. Statistical analysis was performed using two-way ANOVA with a Dunnett’s post-hoc test for multiple comparisons. *n*=4 wells pooled from 2 independent experiments. E, F MANF induces cell migration in transwell assay. HeLa cells migrating through permeable membrane were stained with crystal violet (D). Migrated cell number was represented by OD570 reading and statistically analyzed using one-way ANOVA with a Dunnett’s post-hoc test for multiple comparisons (E). *n*=3 independent experiments. For all graphs, data are presented as mean ± SD. *: *P*<0.05. **: *P*<0.01. ***: *P*<0.001. ****: *P*<0.0001. Scale bar = 1 mm.

**Additional file 2: Supplementary Figure 2**. KDELR does not bind G_α_ proteins. A Schematic illustration of split-Venus assay. KDELR was fused with N-terminal half of Venus (Halo-KDLR-Flag-VN), while G proteins were fused with C-terminal half of Venus (G-myc-VC). Potential interaction between KDELR and any G protein would generate Venus signals. B HeLa cells were co-transfected with Halo-KDLR-Flag-VN and wildtype G-myc-VC or a mutant mimicking GDP-bound state (G_αs_-S54C-myc-VC, G_αq_-S53C-myc-VC, G_αo_-S47C-myc-VC), and stained with indicated antibodies.

**Additional file 3: Supplementary Figure 3. **A EGFR, TfR, and KDELR were found in CCVs. Cell lysates and CCVs prepared from HeLa cells expressing Halo-KDELR or Halo-KDELR D91A/T92A were analyzed by western blotting with indicated antibodies. B Co-IP experiments in HeLa cells expressing endogenously tagged KDELR-Flag-mCherry using anti-RFP beads confirmed that EGFR and TfR interact with KDELR. C-E HeLa cells transfected with single plasmid were stained with specific antibodies and observed under confocal microscope as negative controls for split Venus assay. Scale bar = 10 μm.

**Additional file 4: Supplementary Figure 4. **A-D KDEL ligand induced the transport of EGFR to the Golgi. HeLa Halo-KDELR and KDELR knockdown cells were treated with DMSO or 50 μM TAEKDEL peptide at 4^o^C for 30 minutes and then incubated at 37^o^C for 0 or 30 minutes. Cells were fixed and stained with anti-TGN46, a Golgi marker, and anti-EGFR antibodies (A-C). Pearson’s coefficient of EGFR and TGF46 at the Golgi was calculated and quantified using two-way ANOVA with a Sidak’s multiple comparisions test (D). *n*=20 cells pooled from 3 independent experiments. Scale bars = 10 μm. ****: *P*<0.0001. E EGF induced the degradation of EGFR. HeLa cells stably overexpressing Halo-KDELR were incubated with 1.5 nM EGF, 200 nM EGF, 50 μM TAEKDEL, or 50 μM TAEAAAA at 37^o^C for 30 minutes. Cell lysates were prepared and analyzed by western blotting. Relative intensities of protein bands were measured using ImageJ and marked under blots.

**Additional file 5: Supplementary Figure 5.** A, B STAT3 inhibitor did not affect the endocytosis of KDELR induced by KDEL ligand. HeLa cells expressing Halo-KDELR were treated with non-permeable HaloTag Alexa Fluor 488 ligand and DMSO or 4 μM cryptotanshinone at 37^o^C for 2 hours, prior to incubation with 50 μM TAEKDEL at 4 ^o^C for 30 minutes. Cells were incubated at 37^o^C for 0, or 30 minutes before staining for IF (A). Pearson’s coefficient of KDELR and clathrin at the Golgi was calculated and quantified using two-way ANOVA with a Sidak’s multiple comparisions test (B). *n*=20 cells pooled from 3 independent experiments. ns=not significant. Scale bar = 10 μm.

## Data Availability

All data need to evaluate the conclusions in this study are present in the paper and/or in the Supplementary Materials.
