# Supplementary figures and images for: Crosstalk between KDEL receptor and EGF receptor mediates cell proliferation and migration via STAT3 signaling
Source: Cell Commun Signal. 2024 Feb 20;22:140. doi: 10.1186/s12964-024-01517-w (PMC10880305; doi:10.1186/s12964-024-01517-w)

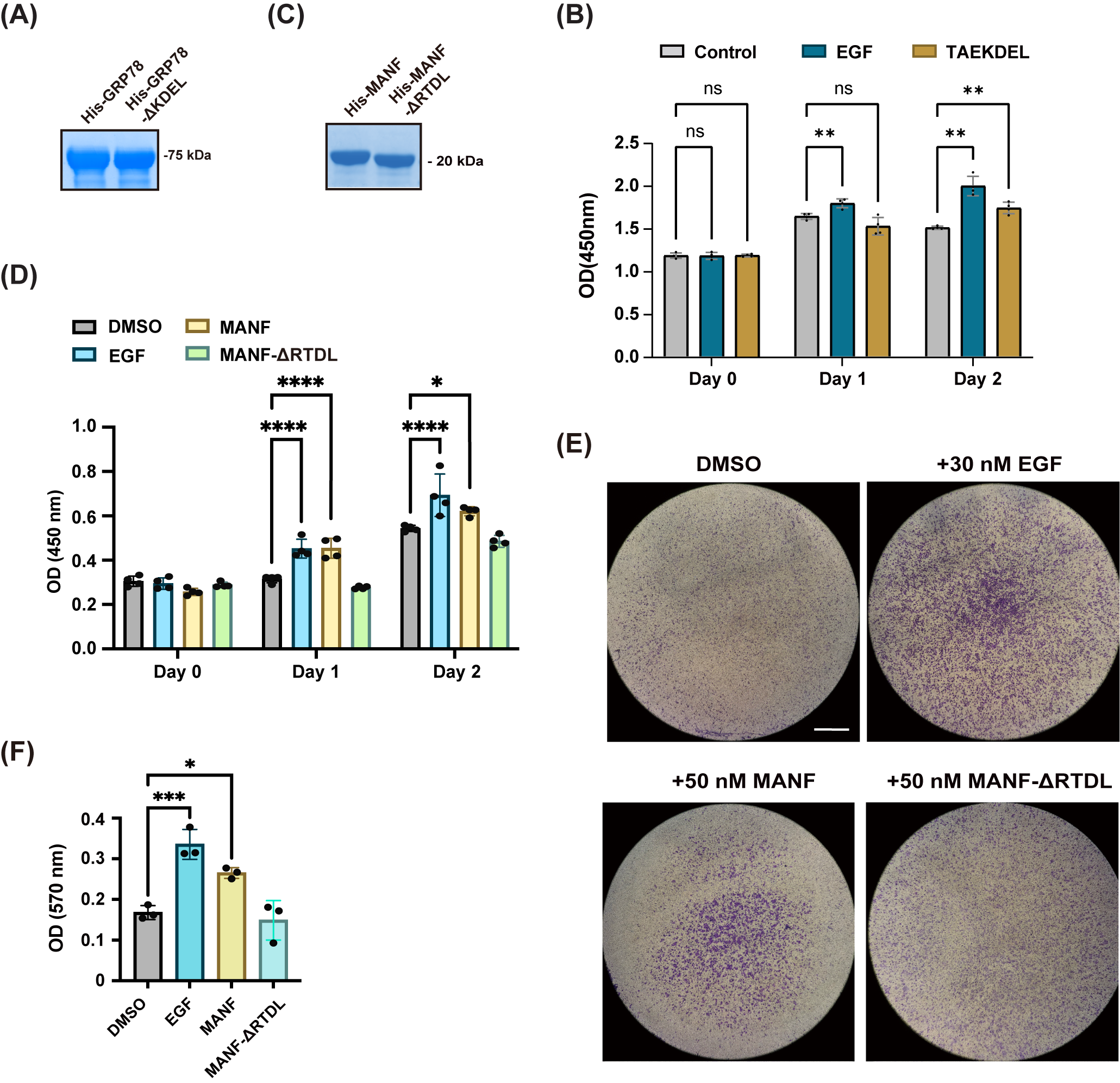

Supplement: Supplementary file 1 — Additional file 1: Supplementary Figure 1. KDEL ligands induce cell growth and migration. A Recombinant His-tagged GRP78 and GRP78ΔKDEL were purified from E. coli and analyzed by Coomassie staining. B TAEKDEL peptide induced cell proliferation. HeLa cells were incubated with DMSO, 10 nM EGF, 50 μM TAEKDEL in DMEM supplemented with 0.1% FBS and subjected to CCK-8 assay on day 0, 1, and 2. Statistical analysis was performed using two-way ANOVA with a Dunnett’s post-hoc test for multiple comparisons. n=3 independent experiments. ns: not significant. **: P<0.01. C His-tagged MANF and MANFΔRTDL were purified from E. coli and analyzed by Coomassie staining. D MANF stimulates cell proliferation. Cell viability was measured by CCK-8 assay on day 0, 1, and 2 after incubation with 30 nM EGF, 50 nM MANF, or 50 nM MANFΔRTDL. Histogram summarized the OD450 measurements of cells at indicated time points. Statistical analysis was performed using two-way ANOVA with a Dunnett’s post-hoc test for multiple comparisons. n=4 wells pooled from 2 independent experiments. E, F MANF induces cell migration in transwell assay. HeLa cells migrating through permeable membrane were stained with crystal violet (D). Migrated cell number was represented by OD570 reading and statistically analyzed using one-way ANOVA with a Dunnett’s post-hoc test for multiple comparisons (E). n=3 independent experiments. For all graphs, data are presented as mean ± SD. *: P<0.05. **: P<0.01. ***: P<0.001. ****: P<0.0001. Scale bar = 1 mm. [file 12964_2024_1517_MOESM1_ESM.tif]

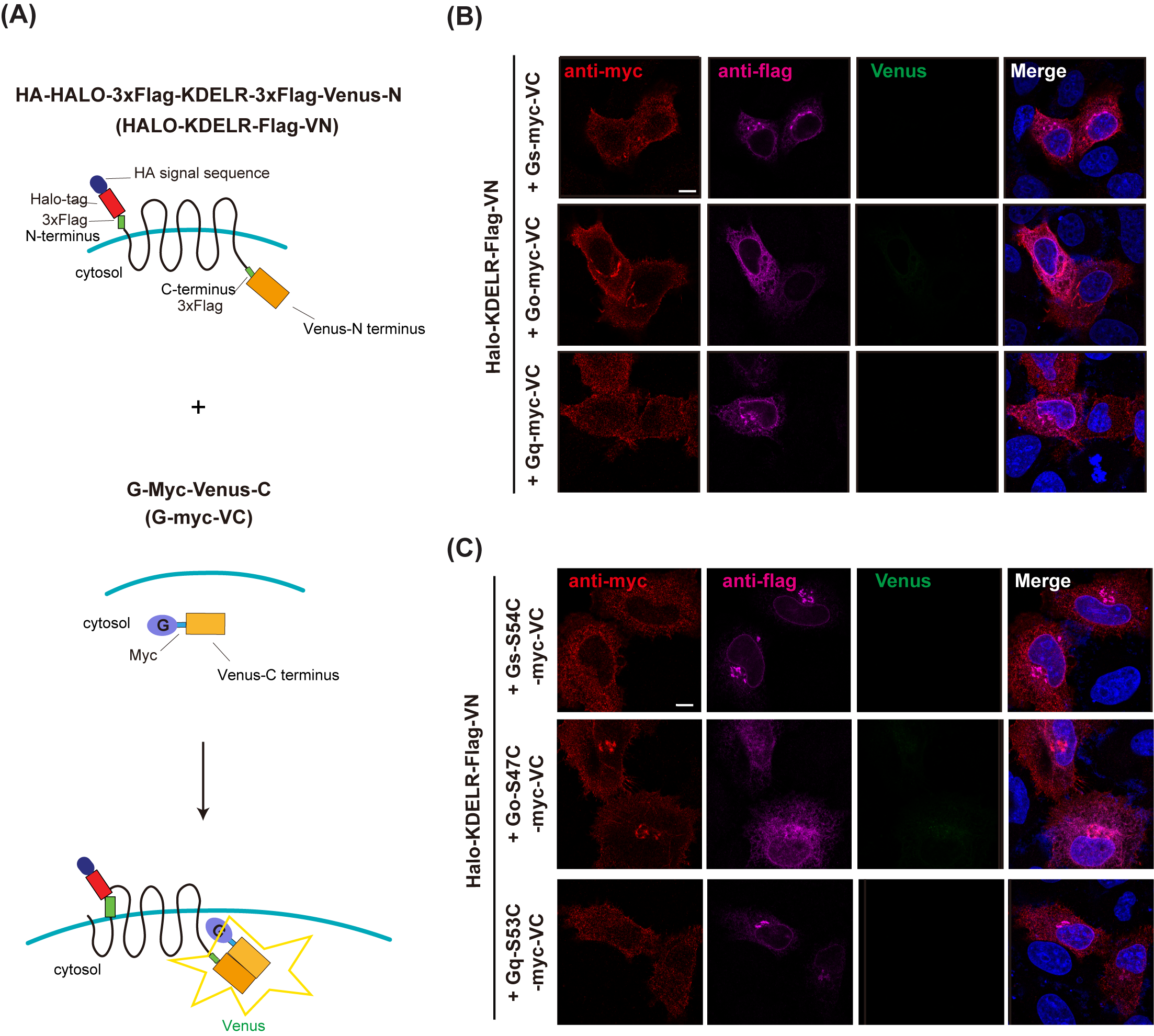

Supplement: Supplementary file 2 — Additional file 2: Supplementary Figure 2. KDELR does not bind Gα proteins. A Schematic illustration of split-Venus assay. KDELR was fused with N-terminal half of Venus (Halo-KDLR-Flag-VN), while G proteins were fused with C-terminal half of Venus (G-myc-VC). Potential interaction between KDELR and any G protein would generate Venus signals. B HeLa cells were co-transfected with Halo-KDLR-Flag-VN and wildtype G-myc-VC or a mutant mimicking GDP-bound state (Gαs-S54C-myc-VC, Gαq-S53C-myc-VC, Gαo-S47C-myc-VC), and stained with indicated antibodies. [file 12964_2024_1517_MOESM2_ESM.tif]

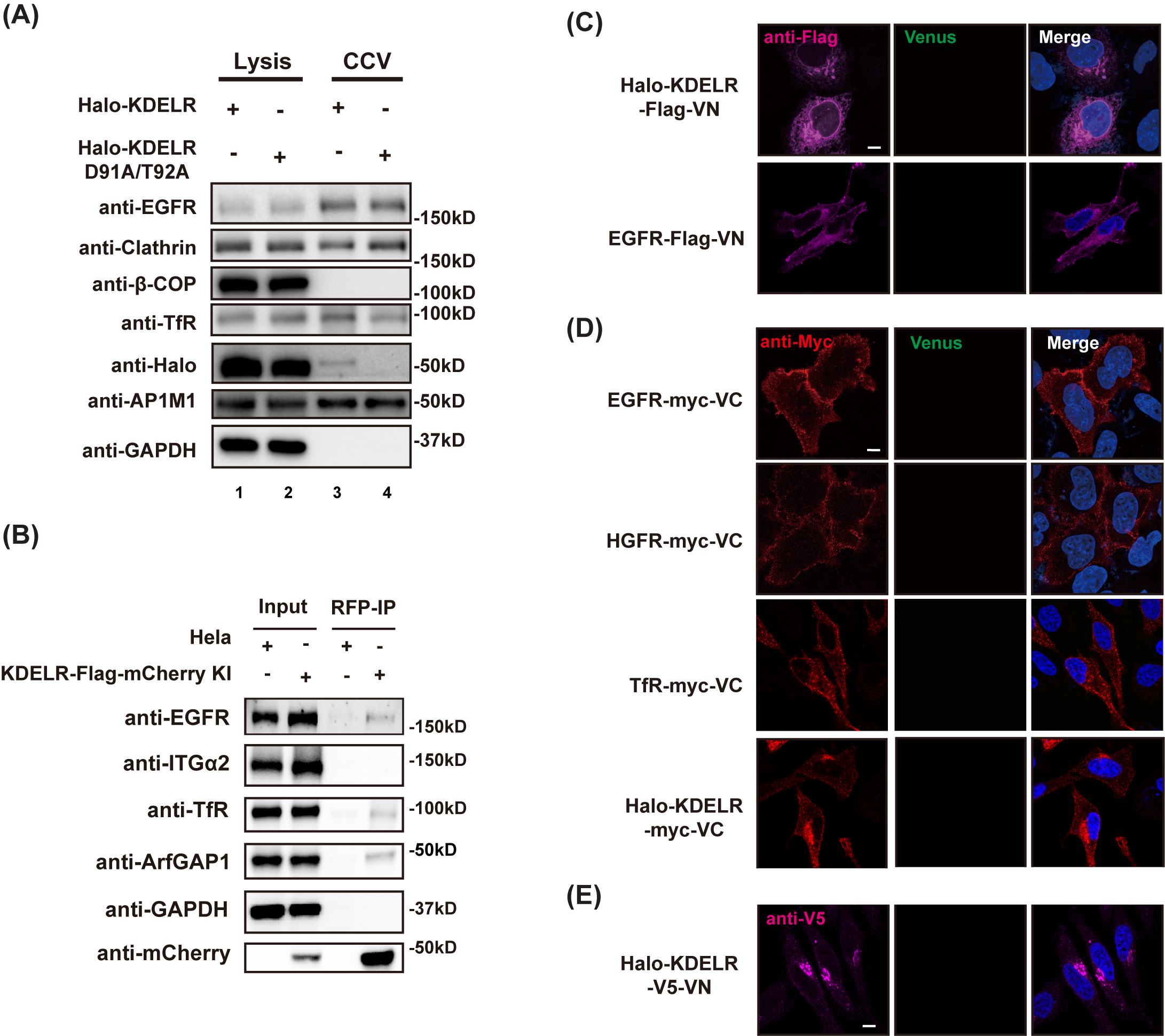

Supplement: Supplementary file 3 — Additional file 3: Supplementary Figure 3. A EGFR, TfR, and KDELR were found in CCVs. Cell lysates and CCVs prepared from HeLa cells expressing Halo-KDELR or Halo-KDELR D91A/T92A were analyzed by western blotting with indicated antibodies. B Co-IP experiments in HeLa cells expressing endogenously tagged KDELR-Flag-mCherry using anti-RFP beads confirmed that EGFR and TfR interact with KDELR. C-E HeLa cells transfected with single plasmid were stained with specific antibodies and observed under confocal microscope as negative controls for split Venus assay. Scale bar = 10 μm. [file 12964_2024_1517_MOESM3_ESM.tif]

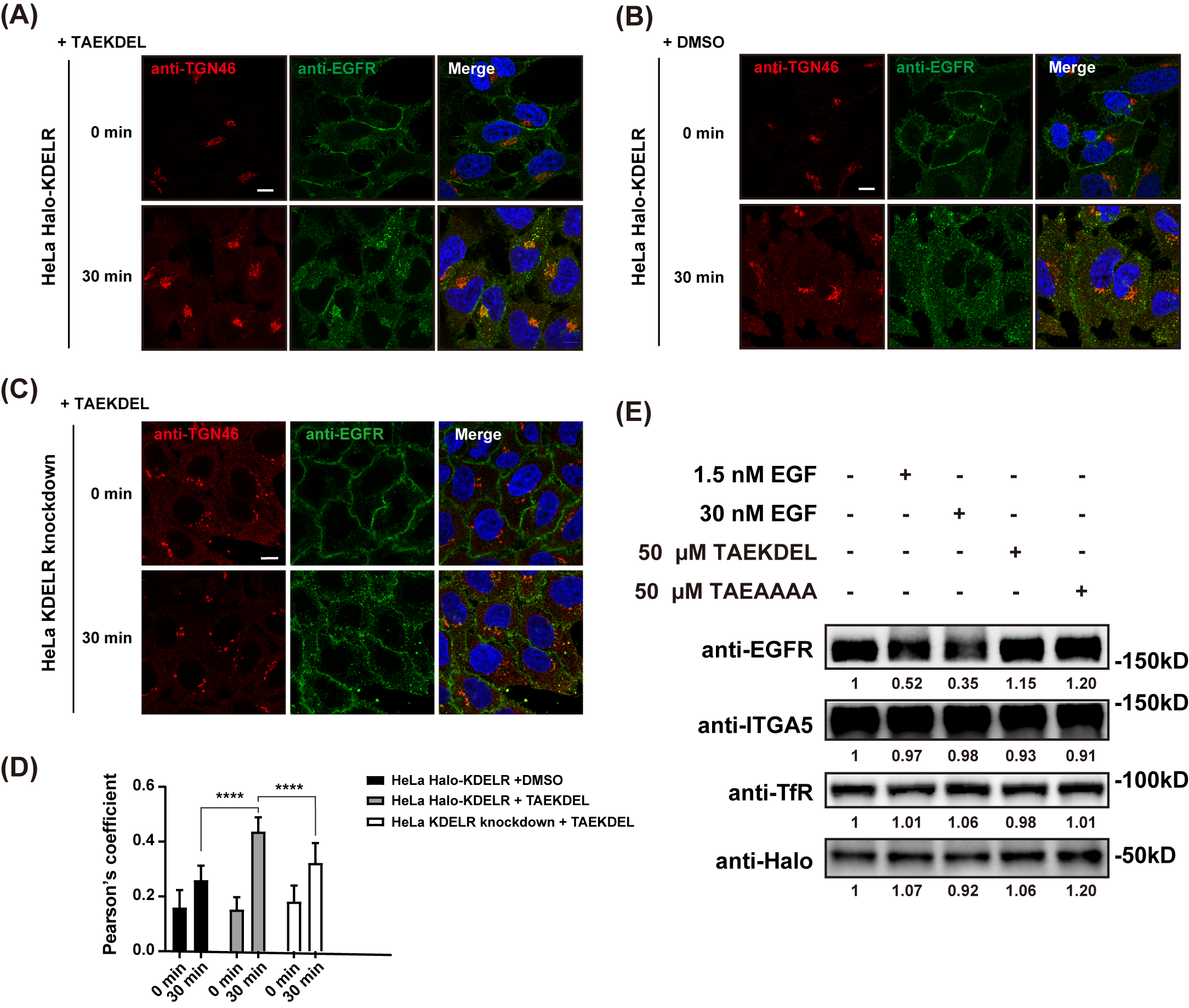

Supplement: Supplementary file 4 — Additional file 4: Supplementary Figure 4. A-D KDEL ligand induced the transport of EGFR to the Golgi. HeLa Halo-KDELR and KDELR knockdown cells were treated with DMSO or 50 μM TAEKDEL peptide at 4oC for 30 minutes and then incubated at 37oC for 0 or 30 minutes. Cells were fixed and stained with anti-TGN46, a Golgi marker, and anti-EGFR antibodies (A-C). Pearson’s coefficient of EGFR and TGF46 at the Golgi was calculated and quantified using two-way ANOVA with a Sidak’s multiple comparisions test (D). n=20 cells pooled from 3 independent experiments. Scale bars = 10 μm. ****: P<0.0001. E EGF induced the degradation of EGFR. HeLa cells stably overexpressing Halo-KDELR were incubated with 1.5 nM EGF, 200 nM EGF, 50 μM TAEKDEL, or 50 μM TAEAAAA at 37oC for 30 minutes. Cell lysates were prepared and analyzed by western blotting. Relative intensities of protein bands were measured using ImageJ and marked under blots. [file 12964_2024_1517_MOESM4_ESM.tif]

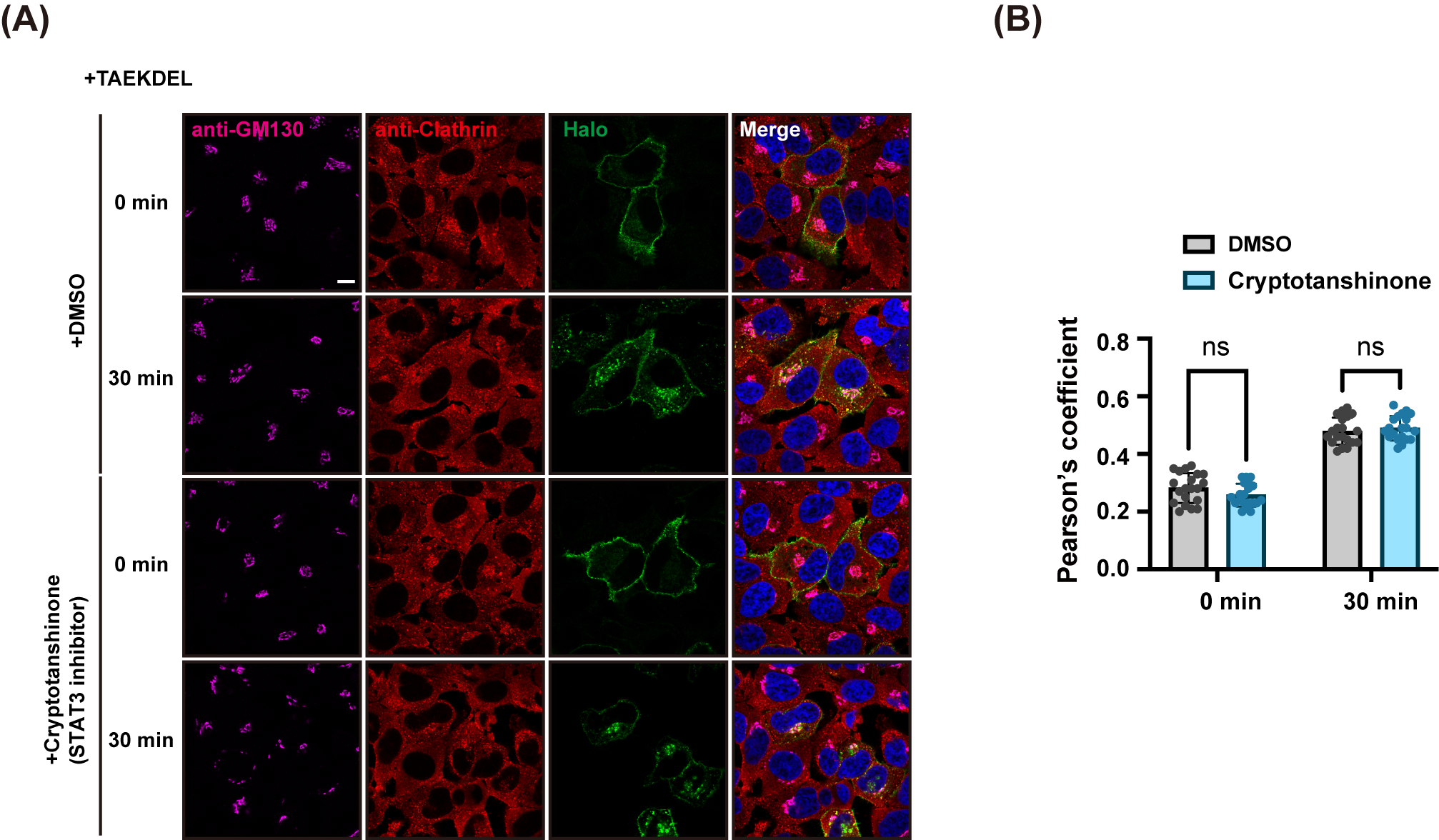

Supplement: Supplementary file 5 — Additional file 5: Supplementary Figure 5. A, B STAT3 inhibitor did not affect the endocytosis of KDELR induced by KDEL ligand. HeLa cells expressing Halo-KDELR were treated with non-permeable HaloTag Alexa Fluor 488 ligand and DMSO or 4 μM cryptotanshinone at 37oC for 2 hours, prior to incubation with 50 μM TAEKDEL at 4 oC for 30 minutes. Cells were incubated at 37oC for 0, or 30 minutes before staining for IF (A). Pearson’s coefficient of KDELR and clathrin at the Golgi was calculated and quantified using two-way ANOVA with a Sidak’s multiple comparisions test (B). n=20 cells pooled from 3 independent experiments. ns=not significant. Scale bar = 10 μm. [file 12964_2024_1517_MOESM5_ESM.tif]
